# An Epigenetic Switch for Sex-Specific Brain Resilience in Stroke: Targeting HDAC2 to Amplify Endogenous Oxytocin Signaling

**DOI:** 10.1021/acscentsci.6c00191

**Published:** 2026-03-25

**Authors:** Nashwa Amin, Xia Yuan, Zongjie Shi, Fei Wu, Irum Naz Abbasi, Yang Yang, Suhong Ye, Qining Yang, Yu Geng, Marong Fang

**Affiliations:** † Department of Orthopedics of Children’s Hospital, Zhejiang University School of Medicine, National Clinical Research Center For Children and Adolescents’ Health and Diseases, 310052 Hangzhou, China; ‡ Institute of System Medicine, 12377 Zhejiang University School of Medicine, Zhejiang University, 310058 Hangzhou, China; § Department of Zoology, Faculty of Science, Aswan University, 81521 Aswan, Egypt; ∥ Center for Rehabilitation Medicine, Department of Neurology, Zhejiang Provincial People’s Hospital, Affiliated People’s Hospital, Hangzhou Medical College, 310014 Hangzhou, China; ⊥ Faculty of Medicine, Macau University of Science and Technology, Macau 999078, Taipa, China; # Department of Neurology and Psychiatry, The Second Hospital of Jinhua, 321004 Jinhua, China; ∇ Department of Orthopaedics, Jinhua Municipal Central Hospital, Affiliated Jinhua Hospital, Zhejiang University School of Medicine, 321000 Jinhua, China

## Abstract

The continuous failure to account for biological sex is a key impediment to developing effective neuroprotective treatments for ischemic stroke. While epigenetic modulators such as HDAC inhibitors show promise, the mechanisms behind their sexually dimorphic effects are unknown. We present a unique, sex-specific mechanism in which HDAC2 suppression offers substantial resilience to ischemic brain injury by significantly increasing the endogenous oxytocin (OXT) signaling axis. Through integrated in vitro and in vivo models, we show that HDAC2 knockdown not only reduces infarct size and enhances functional recovery, but also does so more effectively in females. We attribute this improved protection to a strong, female-specific increase of OXT and its receptor (OTR). This increased OXT signaling, possibly mediated by estrogen, resulted in significant decreases in apoptosis, neuroinflammation, and oxidative stress. Our findings show that HDAC2 serves as a critical epigenetic brake on a built-in neuroprotective mechanism that, when activated, triggers a therapeutically potent, sex-divergent response. This study sheds light on chemical biology by identifying a druggable epigenetic target that modulates an important neurohormonal circuit. More broadly, it establishes a new paradigm for individualized stroke therapy, shifting away from a one-size-fits-all strategy and toward leveraging innate, sex-specific protective mechanisms to improve treatment efficacy.

## Graphical abstract

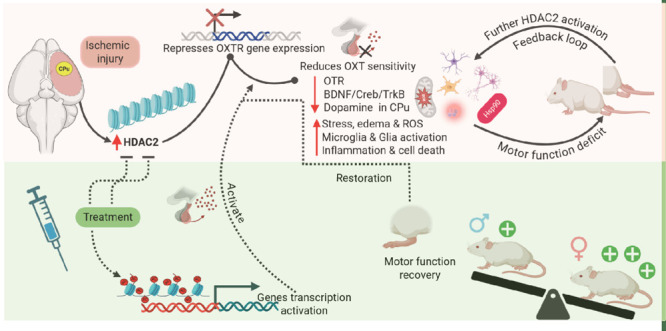

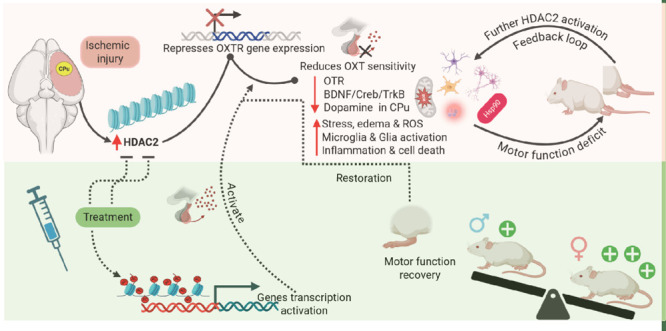

## 1 Introduction

Stroke, predominantly ischemic in nature and constituting approximately 85% of cases, is a significant global contributor to mortality and disability. Notwithstanding progress in acute stroke treatment, including thrombolysis and thrombectomy, numerous patients still experience enduring neurological deficits due to the absence of efficacious neuroprotective and neurorestorative medications.^1^ Recent studies have focused on the molecular processes that underpin stroke causation and recovery, with a particular emphasis on epigenetic regulation and neuropeptide signaling. Histone deacetylases (HDACs), which regulate gene expression by chromatin remodeling, play a significant function in this scenario. Specifically, HDAC2, a class I HDAC, has been linked to synaptic plasticity, neuroinflammation, and neuronal survival. Overexpression of HDAC2 in stroke has been linked to impaired functional recovery, decreased neurogenesis, and neuronal death.^2^ Preclinical experiments have shown that inhibiting HDAC2 may increase neuroprotection and recovery, indicating that it might be a therapeutic target.^3^ However, the role of HDAC2 in sex-specific stroke outcomes is unknown. Oxytocin, a neuropeptide with known effects in social behavior and reproduction, has emerged as a potential neuroprotective agent in stroke. In animal models, oxytocin has been found to decrease infarct volume, reduce neuroinflammation, and increase neurogenesis and angiogenesis. These benefits are delivered via anti-inflammatory, antioxidant, and synaptic plasticity-enhancing mechanisms. Furthermore, oxytocin has been shown to influence epigenetic processes such as histone acetylation, which may relate it to HDAC2.^4^ Men and women respond differently to treatment and have different stroke outcomes, according to current clinical studies.^5^ Women often have inferior functional results and higher death rates as a result of hormonal, genetic, and societal influences. For example, whereas estrogen has neuroprotective qualities, its reduction after menopause may worsen stroke severity in older women.^6^ Although sex variations in HDAC2 expression and oxytocin signaling have been identified, their influence on stroke recovery is not well characterized.

HDAC2, a member of the histone deacetylase family, removes acetyl groups from histone proteins, causing chromatin condensation and transcriptional repression. This epigenetic change may influence the expression of genes involved in neuroprotection, inflammation, and cell survival.^7^ (Scheme 1A). In ischemic stroke, HDAC2 controls pro-inflammatory gene expression, and high HDAC2 activity has been associated with increased neuroinflammation and brain damage.^8^ Moreover, HDAC2 influences synaptic plasticity and cognitive functions; its inactivation has been shown to enhance synaptic plasticity and cognitive recovery following an ischemic stroke.^9^ Due to its involvement in gene repression and neuroinflammation, HDAC2 is seen as a promising therapeutic target in ischemic stroke. The neuroprotective characteristics of HDAC2 inhibitors in preclinical animal studies render them an intriguing candidate for future investigation.^10^ Research on animals has demonstrated that pharmacological or genetic inhibition of HDAC2 may diminish neuroinflammation, decrease infarct size, and enhance functional recovery. Nonetheless, additional research is necessary to comprehensively elucidate these pathways and develop clinically effective HDAC2 inhibitors.

**Scheme 1:**
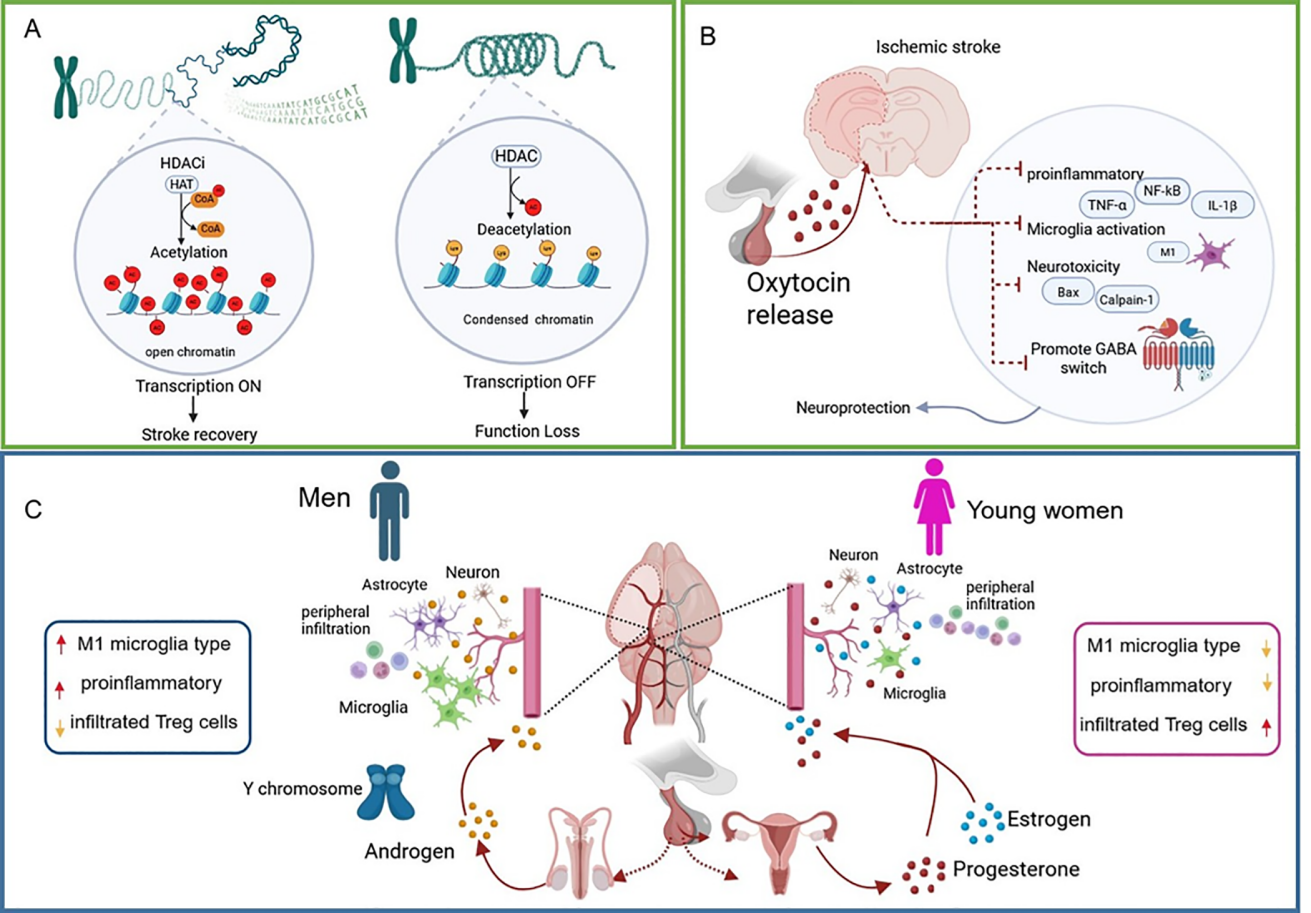
[Fn sch1-fn1]

a
**A**; Gene transcriptional activity is influenced by HATs’ acetylation and HDACs’ deacetylation. By adding or removing acetyl groups at the N–terminus lysine, HATs and HDACs cause the chromatin to become open or compacted. By reversing the reduced peri-infarct cortical neuroplasticity, HDACis epigenetically stimulate the expression of neuroplasticity-related genes to improve functional recovery following stroke. HDAC2 means histone deacetylase 2; HDACis, histone deacetylase inhibitors. **B**; OXT attenuates ischemic brain injury via inhibiting apoptosis, suppressing inflammation (TNF-α and IL-1β) by inhibiting the expression of NF-κB. These are beneficial for the recovery of cognitive function after an ischemic stroke. In addition, OT attenuated neurotoxicity, which may be associated with the inhibition of NF-κB. OXT promotes the GABA switch. **C**; Sex hormones, genetics and epigenetics, and immunological reactions promote different responses to stroke dependently or collaboratively between the sexes.

Oxytocin (OXT), a neuropeptide formerly connected with social bonding, childbirth, and breastfeeding, has lately received interest for its possible neuroprotective and anti-inflammatory actions in ischemic stroke (Scheme 1B). OXT reduces oxidative stress by scavenging free radicals and increasing antioxidant enzyme activity, sparing neurons from damage.^11^ OXT may decrease neuroinflammation by decreasing pro-inflammatory cytokines including TNF-α and IL-6 and increasing anti-inflammatory responses, potentially limiting secondary brain damage.^12^ Furthermore, OXT improves the blood–brain barrier (BBB), which is often compromised in ischemic stroke. Preserving BBB integrity helps to minimize cerebral edema and prevent hazardous chemicals from entering the brain.^13^ Importantly, OXT may improve synaptic plasticity and neurogenesis, hence accelerating poststroke recovery. It also inhibits the caspase-3 pathway, which prevents neuronal death while increasing brain repair.^14^ OXT’s vasodilatory qualities may enhance cerebral blood flow to ischemic areas, thereby lowering the severity of damage. Furthermore, OXT controls glutamate signaling, which reduces excitotoxicity, a major contributor to neuronal death in stroke.^15^ Its capacity to reduce stress and anxiety may indirectly aid in recovery by increasing mental health and rehabilitation adherence.

The oxytocinergic system is increasingly recognized as a key regulator of the stress response. Critically, preclinical models reveal that oxytocin’s impact on stress responsivity is not uniform but is sex-dependent, with robust anxiolytic effects frequently observed in males and more complex, hormonally modulated effects in females. While historically explored in male-biased rodent studies, the oxytocin-stress axis is now being actively translated to human research, where emerging evidence confirms significant sexual dimorphism in its behavioral and neuroendocrine effects, underscoring sex as a crucial variable for understanding its therapeutic potential.^16^

Ischemic stroke has substantial sex differences in epidemiology, risk factors, clinical presentation, and prognosis, which are impacted by biological, hormonal, and social variables. Understanding these distinctions is crucial for creating focused preventative measures, increasing diagnostic accuracy, and optimizing treatments for both men and women.^17^ Sex and age have a substantial impact on stroke incidence: males have a greater risk at earlier ages, but women have a larger lifetime risk owing to their longer life expectancy.^18^ Postmenopausal women had a much higher incidence of stroke than males, most likely owing to the loss of estrogen’s vascular protective properties.^19^ Sex-specific risk factors exacerbate these discrepancies. Pregnancy-related disorders (e.g., pre-eclampsia, gestational diabetes) and oral contraceptive usage increase the risk of stroke in women,^20^ whereas postmenopausal hormonal changes make them more vulnerable. Men, on the other hand, are more likely to have risk factors such as atrial fibrillation, smoking, and excessive alcohol intake, which contributes to their increased stroke incidence at younger ages.^21^ Women typically come with atypical stroke symptoms (e.g., weariness, confusion), which causes delays in diagnosis and treatment,^22^ while males are more likely to display classic symptoms such as hemiparesis or speech problems.^23^ Women had lower functional outcomes and higher death rates after stroke, which might be attributed to their older age at start, larger stroke severity, and higher comorbidity load.^24^ Social issues, such as living alone or in care facilities, may also restrict their rehabilitation options. Despite a greater recurrence risk, males frequently recover faster, perhaps owing to earlier diagnosis and more intensive therapy.^25^ Estrogen’s vasoprotective actions account for premenopausal women’s decreased stroke risk compared to age-matched males.^26^ However, in postmenopausal women, hormone replacement treatment (HRT) has been linked to an increased stroke risk, emphasizing the complexities of hormonal impacts.^27^ More study is required to better understand these pathways and create safer therapy techniques.

Sociocultural variables also influence sex inequalities in stroke outcomes. Women are more likely to live alone after a stroke, which may restrict access to rehabilitation, while males may get more intense treatment owing to cultural expectations.^28^ These variations highlight the need for Sex-sensitive stroke care.^29^ In conclusion, sex differences in ischemic stroke are complex, encompassing biological, hormonal, and social aspects (Scheme 1C). While males are more likely to suffer a stroke at a younger age and recover more quickly,^30,31^ women have a larger lifetime risk, worse functional results, and higher death rates. Understanding these variances is critical for creating sex-specific preventive, diagnostic, and treatment plans. Future studies should look at the underlying processes and consider targeted remedies. The putative interaction of oxytocin (OXT) with histone deacetylase 2 (HDAC2) is becoming more interesting. Although there is presently no direct evidence connecting HDAC2 and OXT, both molecules have important roles in regulating social behavior, brain development, and gene expression, indicating a potential relationship.^32^ This research will look at the link between HDAC2 and oxytocin in stroke recovery to better understand how their interaction affects neuroprotection, neurogenesis, and functional results in both men and women. In this study, we look at differences based on biological sex, gonadal, and hormonal traits, not gender identity, which is achieved by combining genetic, cellular, and behavioral approaches to discover sex-specific therapeutic targets and find new mechanisms driving stroke recovery.

## 2 Materials and Methods

### 2.1 In Vitro

#### 2.1.1 Cell Culture of SH-SY5Y and HT-22 Cells

Female-derived human neuroblastoma (SH-SY5Y) cells and male-derived mouse hippocampal (HT-22) neurons cell line serve as a valuable in vitro model for studying neuronal development and function in neuroscience research. For cultivation, Cells were maintained in an environment of 5% CO_2_/95% air at 37 °C, using DMEM/F12 (1:1, v/v) medium supplemented with 10% FBS (Invitrogen), 2 mM l-glutamine, and 1% penicillin/streptomycin (Invitrogen).

#### 2.1.2 Establishment of the OGD/R Model and Treatment

To induce oxygen-glucose deprivation (OGD) in vitro, cells were first washed twice with preheated PBS and then plated in a preheated, glucose-free RPMI-1640 medium (Gibco) at a density of approximately 3 × 10^4^ cells per well. The plates were placed in an anaerobic culture bag containing an anaerobic gas-producing bag and an anaerobic indicator (Mitsubishi Gas Chemical, Japan), and incubated for 6 h in a CO_2_ incubator. After removing the anaerobic apparatus, the cells were allowed to recover in complete culture media with apicidin (0.2 μM, Sigma-Aldrich, 17827), a potent and cell-permeable histone deacetylase II inhibitor, for 24 h hours at 37 °C with 5% CO_2_. The experimental groups included: HT-22: Normal cells, OGD-treated cells, and OGD-treated cells with HDAC2.i (apicidin), SH-SY5Y: Normal cells, OGD-treated cells, and OGD-treated cells with HDAC2.i (apicidin) (Scheme.[Fig sch2]).

**Scheme 2:**
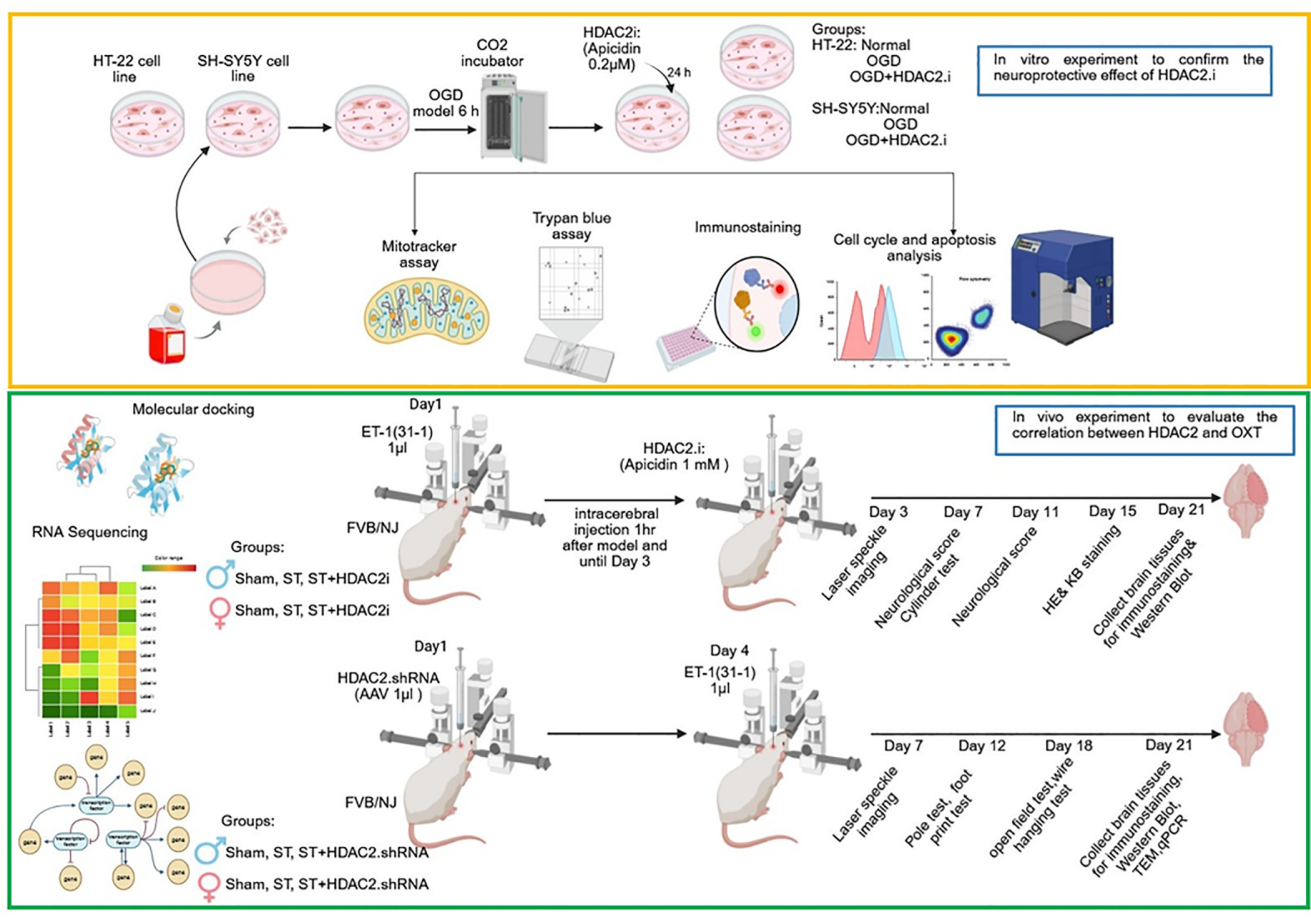
Experimental Study Plan^a^

a Preliminary *in vitro* experiment was established to confirm the protective effect of HDAC2 inhibition by using flow cytometer, *in vivo* experiment by using pharmacological HDAC2 inhibition and HDAC2 knockdown.

#### 2.1.3 Trypan Blue Assay

The trypan blue assay was used to assess cell viability by staining dead or damaged cells with compromised membranes. A 0.4% trypan blue solution was prepared in a buffered isotonic salt solution (e.g., phosphate-buffered saline, pH 7.2 to 7.3. Equal volumes of the cell suspension and trypan blue solution were mixed and incubated for 2 min at room temperature. Cells were counted using a hemocytometer under a microscope at low magnification, distinguishing live (unstained) and from dead (blue-stained) cells. Cell viability was calculated as

%CellViability=(TotalNumberofCells−NumberofDeadCells)/TotalNumberofCells×100

#### 2.1.4 Detection of Apoptosis by Flow Cytometry Analysis

After 48 h of incubation in a CO_2_ incubator, cells were trypsinized using 0.25% trypsin and adjusted to a density of 1 × 10^6^ cells/mL. Apoptosis was detected using the PE Annexin-V Apoptosis Detection Kit I (BD Pharmingen, USA) and CytoFLEX LX flow cytometry (Beckman Coulter, Brea, CA, USA). Cells were resuspended in 100 μL of 1× annexin-binding buffer, stained with 5 μL PE Annexin-V and 7-AAD in the dark for 15 min at room temperature, and then diluted with 400 μL of 1× annexin-binding buffer before flow cytometry analysis. The CytExpert program was used to quantify the rate of dead cells.

#### 2.1.5 Cell Cycle Evaluation by Flow Cytometry Analysis

The cell cycle was analyzed using PI/RNase Staining Buffer (BD Pharmingen, Cat. No. 550825). After fixation and permeabilization, 0.5 mL of cell suspension (1 × 10^6^ cells) was incubated for 15 min at room temperature. Cells were washed by centrifugation at 1,000 rpm for 10 min, and supernatant was aspirated. Fixation was carried out at 4 °C for 15–30 min. The pellet was vortexed, and 5 mL of cool 70% to 80% ethanol was added dropwise. Then washed thrice with 1X PBS and Stain Buffer. For staining, 10^6^ cells were resuspended in 0.5 mL PI/RNase Staining Buffer for PI/RNase staining. Alternatively, the cells were resuspended in 0.1 mL of Stain Buffer or 0.1 mL Staining Buffer for PI/RNase staining. Alternatively, the cells were resuspended in 0.1 mL of Stain Buffer with 20 μL of 7-AAD Staining Solution, incubated for 15 min at room temperature, and analyzed. Within 1 h by flow cytometer.

#### 2.1.6 Mitochondrial Probe Assay

The cells were plated on glass coverslips in 6-well plates, and treated with oxygen-glucose deprivation (OGD) and the HDAC2 inhibitor (Apicidin). Coverslip were stained with Mitotracker (1:5000 dilution,1 mM stock) for 30 min at 37 °C, washed with PBS, fixed with 3.2% paraformaldehyde for 20 min at room temperature). Coverslips were mounted on slides, stored at 4 °C, and imaged using an Olympus FV120 laser-scanning confocal microscope. ImageJ Pro Plus software was utilized to assess the intensity of five randomly selected areas of each segment.

#### 2.1.7 Immunofluorescence Staining

Cells on coverslips were fixed with 4% paraformaldehyde for 40 min at room temperature, permeabilized with 0.2% Triton X-100 in PBS for 15 min, and blocked with 5% BSA. Primary antibody (rabbit anti-MAP2, 1:500) was applied overnight at 4 °C, followed by secondary antibody (antirabbit Alexa Fluor 594, 1:500, EARTHOX) for 1.5 h at room temperature. Slides were mounted with DAPI-containing medium (VECTASHIELD) and imaged at 200× magnification using an Olympus FV120 microscope. Five fields per slide were analyzed with ImageJ.

### 2.2 In Vivo

#### 2.2.1 Animals

Male and female FVB/NJ mice, weighting approximately 28–30 g and aged 3–6 months, were included in this study. Mice exhibiting inconsistent body weight or abnormal behavior were excluded. All mice were housed in the animal center under a 12 h/12 h light cycle, with room temperature maintained at 24 °C and humidity at 40%–60%. Food and water were provided ad libitum. Prior to experimentation, the animals were transported to the laboratory (Scheme.[Fig sch2]). FVB mice are suitable for creating models of focal cerebral ischemia, as a single injection of ET-1/L-NAME induces a larger lesion compared to other strains. All animal procedures were approved by the Laboratory Animal Center of Zhejiang University and complied with relevant ethical regulations

#### 2.2.2 Intracranial Injection of ET-1 (1–31)

ET-1 (1–31) was intracranially injected using a mouse stereotaxic instrument, an established method for simulating ischemic stroke with low mortality. ET-1 (1–31) was dissolved in L-NAME solution for optimal effect. Following disinfection, a 1 cm scalp incision was made along the midline between the eyes and ears. The skull was surgically exposed according to the mouse brain atlas, targeting the area controlling the right forelimb motor cortex. The injection site coordinates relative to bregma were: +0.8 mm AP, −1.5 mm ML, and +2.5 mm DV. After needle insertion, a 5 min interval preceded ET-1 injection, and the syringe was retained for an additional 5 min postinjection to prevent backflow. A syringe pump with a 2.5 μL Hamilton syringe was used to inject 1 μg of ET-1 in 1 μL of PBS (400 pmol/μL) at a rate of 0.2 μL/min. ET-1(1–31) (400 pmol in 1 μL) was coinjected with TRITC-dextran (0.5% w/v) to verify consistent delivery between groups. TRITC fluorescence was quantified at the injection site (ex/em: 557/576 nm). All fluorescence values were normalized to the group mean to control for injection variability

#### 2.2.3 TTC Staining

The mouse was sacrificed 24 and 72 h after the operation. The changes in infarction area in both sexes were measured by the TTC staining method. Take fresh brains, store them at 20 °C for 30 min, quickly cut them into 2 mm coronal slices, place the slices in 2% TTC solution (BL1215A), and incubate them in the dark at 37 °C for 20 min. Then fix the slices with 4% PFA at room temperature for 1 h. The infarct area was measured using ImageJ software. The infarct area is calculated by dividing the infarct area of the ipsilateral hemisphere by the total area.

#### 2.2.4 ELISA

The baseline concentrations of NeuN, GFAP, Bax, and Bcl2 in the cortex of the mice were detected by specific ELISA kits [all from MEIMIAN, China] 24 h after model. After eliminating the background, the optical density at 450 nm was determined, and a standard curve was drawn. Experimental procedures were done based on the instructions in the corresponding kit.

#### 2.2.5 Ovariectomy (OVX) Surgical Model, Hormonal and Pharmacological Manipulations

To investigate our mechanistic claims about estrogen-mediated neuroprotection. We made a pre-experiment based on ovariectomy, hormonal and pharmacological manipulations. Female mice were subjected to bilateral ovariectomy (OVX group) to establish an estrogen-deficient state. Briefly, animals were anesthetized with anesthetic, 2% isoflurane, and a single dorsal midline incision was made. The ovarian bursa was located, the oviduct was ligated, and the ovaries were excised. The muscle layer and skin were sutured separately. Sham-operated controls underwent identical procedures without ovary removal. All animals were allowed to recover for 1 week prior to the start of experimental treatments. All drugs were prepared fresh daily in the appropriate vehicle (sesame oil for steroids, sterile saline for peptides and inhibitors). 17β-Estradiol (ES) (HY-B0141): OVX+ES mice received subcutaneous injections of 17β-estradiol (0.2 mg/kg) for 7 consecutive days. Aromatase Inhibitor Exemestane (HY-13632): Female mice received daily intraperitoneal (i.p.) injections (50 mg/kg) 7 days to deplete endogenous estrogen. Oxytocin Receptor Antagonist (OTR antagonism): Animals in the Male and Female received an i.p. or subcutaneous injection of a selective OTR antagonist SHR1653 (HY-128351) at 10 mg/kg 1 h prior to stroke induction. Oxytocin Release Suppressor ((−)-U-50488 hydrochloride, HY-1500830): Female received an i.p. injection (10 mg/kg) 30 min prior to stroke induction. All drugs injection time point are prior to stroke induction and behavioral testing. All corresponding control groups received equivalent volumes of the respective vehicle at the same time points.

#### 2.2.6 Estrous Cycle Monitoring

Vaginal cytology was performed to monitor the estrous cycle stage in all female mice. Beginning 14 days prior to experimental procedures and continuing until the end point, a daily vaginal lavage was performed between 08:00 and 10:00 using 20 μL of sterile 0.9% saline. The lavage fluid was immediately applied to a glass slide, air-dried, and fixed with 100% methanol for 5 min. Slides were then stained with a 0.1% methylene blue solution (61–73–4) for 30 s, rinsed gently with distilled water, and air-dried. Estrous stage was determined by microscopic examination (10×) of the predominant cell types: proestrus (primarily nucleated epithelial cells), estrus (abundant anucleated, cornified squamous epithelial cells), metestrus (approximately equal mix of cornified cells and leukocytes), and diestrus (predominantly leukocytes). For all primary end point experiments involving stroke surgery, behavioral testing, and tissue collection, we exclusively used female mice in the proestrus stage. Female mice in other stages (estrus, metestrus, diestrus) were scheduled for procedures on subsequent days once they entered proestrus.

#### 2.2.7 Pharmacological Intervention

Apicidin, a histone deacetylase inhibitor­(HDAC2.i), was administered to evaluate its effects on stroke recovery and potential sex differences. The stock solution (10 mM) was prepared by dissolving 6.24 mg Apicidin (A8851, Sigma-Aldrich) in 1 mL DMSO, followed by vortexing (10–20 s) and brief sonicated (30–60 s). To ensure complete dissolution, and stored at −80 °C for long-term storage. The working solution (1 mM) was diluted in aCSF. Two μL Apicidin was injected intracerebrally 1 h after model at the same target site and continued for 2 days. To confirm equivalent biological engagement of the target, we assessed a downstream molecular marker histone H3 acetylation in the PFC in a separate cohort 2 h postinjection. On day 21 animals were sacrificed for brain tissue collection. Groups were designed as Male: Sham, St, St+HDAC2.i; Female: Sham, St, St+HDAC2.i (Scheme 2).

#### 2.2.8 HDAC2 Knockdown Virus Construction and Injection

To suppress gene expression of HDAC2, AAV9 vector (adeno-associated virus serotype 9: pAAV-U6-shRNA­(Hdac2)-CMV-EGFP-F2A-Puro-WPRE, 9.83 × 10^12^ V.G./mL; control (pAAV-U6-shRNA-CMV-EGFP-F2A-Puro-WPRE), 6.70 × 10^12^ V.G./mL; Obio Technology­(Shanghai, China)) were used. Viruses were injected stereotaxically 3 days before ET-1 administration using a 2.5 μL Hamilton syringe (Hamilton, Nevada, USA) and Syringe pumps (KD Scientific, 78–8130, USA) (0.2 μL/min, 1 μL total). To quantify transduction, we analyzed GFP+ cell counts and fluorescence intensity in the target region (mPFC) in a subset of mice sacrificed at the 3-week postinjection time point. Groups were: Male - Sham, St, St+HDAC2.shRNA; Female - Sham, St, St+HDAC2.shRNA (Scheme 2).

#### 2.2.9 Laser Speckle Imaging

Cerebral blood flow was assessed 72 h post-ET-1 injection using laser speckle contrast imaging (LSCI; RWD). A 0.5 × 0.5 mm cortical area was scanned at 4.4 Hz with 5 ms exposure, 1 s time constant, and 2.2× magnification. Data were analyzed over 10 s (2048 × 2048 resolution, 10 frames).

#### 2.2.10 Molecular Docking

Molecular docking was performed using Auto Dock Vina software. Small molecules and proteins were preprocessed, with the active site defined as the entire protein. The docking center coordinates were set to center_x = −3.9, center_y = 1.3, and center_z = −0.3. The box size was conFigureured as a cube with a side length of 90 Å, and the spacing step was set to 0.375. Conformational sampling and scoring were carried out using a genetic algorithm. The docking results were ranked based on docking scores, and the optimal conformation was selected for binding mode analysis. Results were analyzed and visualized using PyMOL software (Version 3.0.3). Small molecule-protein interactions included Apicidin-OXT, Apicidin-HDAC2, Apicidin-Bax, and Apicidin-TNF-α. Protein–protein interactions included HDAC2-OXT, HDAC2-OTR, OXT-H3Lys27, and OXT-H3Lys9.

#### 2.2.11 RNA-seq Analysis

RNA-seq analysis and library were assessed to explore the potential expressed genes after stroke in both male and female. At the end of the experiment, Animals (*n* = 3 for each group) were sacrificed and cortex tissue samples from each group were dissected and subjected to total RNA extraction using TRIzol reagent (Invitrogen, Carlsbad, CA) according to the manufacturer’s instruction. RNA quality was assessed using an Agilent Bioanalyzer (Agilent Technologies, Palo Alto, CA). The RNA was sheared and reverse transcribed using random primers to obtain cDNA, which was used for library construction. Illuminam RNA-Seq libraries were subsequently performed using the SMARTer Stranded RNASeq Kit (Clontech, Mountain View, CA) according to the manufacturer’s instructions. All statistical analyses were carried out with HTSeq v0.6.1; after removing transcriptionally inactive genes (read count per million <1) from raw RNA sequencing gene counts, we obtained high-confidence gene counts. For gene expression analysis, the matched reads were calculated and then normalized to reads per kilobase of exon model per million mapped reads (FPKM). The RNA-seq data of raw and processed files have been deposited in NCBI Gene Expression Omnibus, accession number GSE164960.

#### 2.2.12 Functional Enrichment Analysis and Construction of the PPI Network for DEGs

The enrichment analyses using GO terms (http://www.geneontology.org/) and KEGG pathways (http://www.genome.jp/kegg/) data for gene sets were performed using the NIH Database for Annotation, Visualization, and Integrated Discovery (DAVID) web tool. This tool can provide a functional interpretation of huge gene lists derived from genomic studies. A Benjamini *p*-value of < 0.05 was used in the analysis. The PPI network for 106 DEGs, as determined by the STRING database (https://string-db.org/), consisted of 97 nodes and 145 interactions.

#### 2.2.13 Hematoxylin-Eosin (HE) and Kluver-Barrera (KB) Staining

Hematoxylin-eosin (HE) and Kluver-Barrera (KB) staining were used to evaluate postischemic morphological changes. KB staining combines luxol fast blue (for myelin visualization) with cresyl violet (for neuronal nissl substance staining), enabling simultaneous assessment of myelination and neuronal integrity. Staining procedures were performed according to standard protocols. Hematoxylin solution was used to stain transverse slices of 7-μm-thick for 6 min, dipped into acidic ethanol (1% HCl in 70% ethanol) for several seconds, and then washed with distilled water for 10 min after deparaffinization and rehydration. The sections were then stained with eosin solution for 5 min, dehydrated using a graded alcohol series, and cleaned in xylene. The slides were sealed with neutral balsam then ready for microscope examination. Likewise, KB staining was performed on 7-μm-thick transverse sections. The procedure begins with deparaffinization of formalin-fixed, paraffin-embedded (FFPE) sections using xylene (2 changes, 5 min each), followed by rehydration through graded alcohols (100%, 95%, and 70% ethanol, 3 min each) and a distilled water rinse. For myelin staining, sections were immersed in 0.1% Luxol Fast Blue (LFB) solution for overnight at 60 °C or for 2–4 h at room temperature, then rinsed in 95% ethanol to remove excess stain. Then differentiation was performed using 0.05% lithium carbonate solution (10–30 s) followed by 70% ethanol until gray and white matter was verified by microscope. After a distilled water rinse, neuronal staining was achieved using 0.1% Cresyl Violet (5–10 min), followed by brief differentiation in acid alcohol (1% HCl in 70% ethanol, 5–10 s). Sections were then dehydrated through graded alcohols (70%, 95%, and 100% ethanol) and cleared in xylene before mounting with DPX resin.

#### 2.2.14 Neurological Deficit Scoring

Neurological deficit scores were evaluated on postoperative days 3 and 7 following ET-1 injection, graded on a scale of 0–4 (0: no deficit; 4: severe deficit). The scoring criteria were defined as follows: 0 denotes no deficit was noticed; 1 represents forelimb flexion; 2 represents forelimb flexion along with a decreased lateral push resistance; 3 represents unidirectional encirclement; and 4 represents unidirectional encirclement paired with a decreased conscious level. All behavioral observations and statistical analyses were performed under a double-blinded condition.

#### 2.2.15 Open Field Test

The open-field test (OFT) was employed to evaluate locomotor activity and exploratory behavior in mice following the establishment of an ischemic stroke model and subsequent treatment. The instrument dimensions were 45 × 45 × 45 cm, with a white Plexiglas arena associated with an overhead camera and computer. Mice were allowed to explore freely from the center of the arena for 5 min. Total distance, center distance movements, and the number of center entries were recorded and analyzed.

#### 2.2.16 Cylinder Test

The cylinder test assessed spontaneous forelimb asymmetry, particularly in models of unilateral neurological impairment including stroke. Mice were placed inside a glass cylinder (20 cm height, 9–10 cm circumference) and their forelimb contacts during vertical exploration of the cylinder wall were documented. Twenty vertical motions in all were captured and then examined in slow motion by an observer. Data were expressed as a percentage of contralateral (impaired) forelimb relative to total contacts.

#### 2.2.17 Pole Test

The pole test evaluated basic motor coordination. Mice were placed on a vertical shaft (50–55 cm height, 8–10 mm diameter) and two parameters were recorded: (i) total time to descend to the ground (*T*_total_) and (ii) time to complete a 180-degree turn (*T*_turn_). Maximum duration was set to *T*_turn_ and *T*_total_ if an animal fell immediately. If an animal fell laterally without turning, *T*_total_ was linked to *T*_turn_ failure. Trials were invalidated and repeated if the animals paused mid-descent. To prevent climbing, a tiny 7 × 7 cm cardboard square was placed on top of the shaft.

#### 2.2.18 Hanging Test

Grip strength, balance, and endurance were assessed using the wire suspension test. Mice were trained to hang from a horizontal wire (50–60 cm above the ground) between supports. Fall latency served as the primary end point. Masking tape was used sparingly on the back limbs to restrict full-paw usage. A cushion was placed beneath the wire to prevent injury. Focal ischemia can cause sensorimotor failure in the contralateral paw and significantly reduce task performance. Researchers supported mice during training until the mice could sustain their own weight. Animal falls before the end of training will be immediately repositioned on the wire.

#### 2.2.19 Mouse Footprint

Gait and motor coordination were assessed using stride length, base width, and fore-hindlimbs overlap. Footprints were recorded on paper using nontoxic dye and analyzed for anomalies or asymmetries. A 30–50 cm long × 5–10 cm wide walkway was constructed, with white paper fixed to the base. The nontoxic dye (red for forepaws, black for hindpaws) was thin enough to gently cover the mouse’s paws via a shallow tray. Mice trans versed the walkway naturally, leaving footprints for analysis, dye was cleaned off from the paws after testing. High-resolution pictures of the footprints were taken for study by camera once the ink dried. Using a ruler, measurements were taken of the front and rear footprints’ length and breadth, stride length, and distance between prints. Any anomalies or asymmetries that could point to injuries or atypical gait should be noted in the analysis of the footprint pattern.

#### 2.2.20 Western Blotting

Total protein (20 μg) from each sample was separated by electrophoresis on a 15% SDS-PAGE gel at a constant voltage of 200 V for 50 min. Subsequently, proteins were transferred to a PVDF membrane via wet transfer. Then blocked with TBST containing 5% milk for 2 h at room temperature to minimize nonspecific binding. Membranes was incubated with primary antibodies based on recommended dilution (Table S1) overnight at 4 °C. Primary antibodies included: GAPDH (loading control), OXT, OTR, HSP90, BDNF, TrkB, CREB, SYN1, CRP, NSE, P53, Bax, Bcl2, C-Caspase3, TNF-α, iNOS, IL-6, LC3, ATG5, Beclin1, HDAC2, SMN. After extensive washing with TBST, the membrane then incubated with secondary antibody for 2 h at room temperature. Membranes were submitted to hyper-film detection after incubation with the ECL (enhanced chemiluminescence). The grayscale value of each band was quantified using Image Lab software.

#### 2.2.21 Quantitative PCR Analysis

Real-time PCR was conducted to detect RNA expression levels of HDAC2 target genes and OXT signaling-regulating genes, namely, *Hdac2, Hsp90, Smn, Oxt, Otr, Bax, Bcl-2, Caspase3, Tnf-α, Il-1β, Il-6, Il-10, Inos, and Nf-kb*. Real-time PCR-specific primers for mouse β-actin (internal control) were designed using Primer Express software (Table 1). Total RNA from the Cortex tissue was extracted using the Trizol reagent (Invitrogen). All procedures were carried out according to the manufacturer’s instructions. The concentration and purity of RNA samples were determined using Thermo Nanodrop 2000. One microgram of RNA from each sample was reverse-transcribed into cDNA, following the instructions of the DBI-2220 Bestar qPCR RT Kit, using the Bestar 177 SYBR Green qPCR master mix. The reaction program for the PCR was as follows: 50 °C for 2 min, 95 °C for 10 min, followed by 40 cycles of 95 °C for 5 s, 55 °C for 30 s, and 72 °C for 30 s. All mRNA expression levels on the list were detected by RT-PCR. Results were analyzed using the Biorad CFX manager program, version 3.0. All experiments were performed in triplicate.

**Table 1:** 

Gene	Upstream primer	Downstream primer
*Hdac2*	5′-GTTTTGTCAGCTCTCCACGGGT-3′	5′-CTTGGCATGATGTAGTCCTCCAG-3′
*Oxt*	5′-CTGTGCTGGACCTGGATATGCG-3′	5′-AGCTCGTCCGCGCAGCAGATG-3′
*Otr*	5′-TCATCGTGTGCTGGACGCCTTT-3′	5′-GCCCGTGAAGAGCATGTAGATC-3′
*Smn1*	5′-GCCAAAAGGCACAGCCAGAAGA-3′	5′-AGCCGTCTTCTGACCAAACAGC-3′
*Hsp90*	5′-GCTTTCAGAGCTGTTGCGGTAC-3′	5′-AAAGGCGGAGTTAGCAACCTGG-3′
*Il-10*	5′-CGGGAAGACAATAACTGCACCC-3′	5′-CGGTTAGCAGTATGTTGTCCAGC-3′
*Nf-kb*	5′-GCTGCCAAAGAAGGACACGACA-3′	5′-GGCAGGCTATTGCTCATCACAG-3′
*Tnf-α′*	5′-GGTGCCTATGTCTCAGCCTCTT-3′	5′-GCCATAGAACTGATGAGAGGGAG-3′
*Inos*	5′-GTTCTCAGCCCAACAATACAAGA-3′	5′-GTGGACGGGTCGATGTCAC-3′
*Il-1β*	5′-GCAACTGTTCCTGAACTCAACT-3′	5′-ATCTTTTGGGGTCCGTCAACT-3′
*Il-6*	5′-TAGTCCTTCCTACCCCAATTTCC-3′	5′-TTGGTCCTTAGCCACTCCTTC-3′
*Bax*	5′-AAGCTGAGCGAGTGTCTCCGGCG-3′	5′-GCCACAAAGATGGTCACTGTCTGC-3′
*Bcl2*	5′-CTCGTCGCTACCGTCGTGACTTC-3′	5′-CAGATGCCGGTTCAGGTACTCAGT-3′
*Casp-3*	5′-AGCTTCTTCAGAGGCGACTA-3′	5′-GGACACAATACACGGGATCTG-3′
*β-actin*	5′- CTGTCCCTGTATGCCTCTG-3′.	5′- ATGTCACGCACGATTTCC-3′.

#### 2.2.22 Immunofluorescence

Brain tissues were collected after perfusion and fixed in 4% paraformaldehyde. The next day, tissues were immersed in a 30% sucrose solution for cryoprotection. Embedding in OCT compound and sectioning of frozen tissue slices (30-μm-thick) were performed using a freezing microtome (Leica, Wetzlar). Sections were dried at 37 °C, then blocked with 5% normal goat serum in PBS at room temperature for 1 h. Primary antibodies (HDAC2, OXT, OTR, NeuN, AQP4, nNOS, eNOS, Tomm20, Cytochrome C, GFAP, Iba-1, MAP2, NSE, CRP,b-tubulin and HSP90) were applied according to recommended dilution (Table S1) and incubated overnight at 4 °C. The next day, sections were rinsed three times with 0.01 M PBST 5 min per wash. Secondary antibodies (Alexa Fluor antimouse 488 and Alexa Fluor antirabbit 549) were applied and incubated at room temperature for 2.5 h in the dark. After three additional PBST washes (5 min each), the slides were coated with a DAPI-containing mounting solution (VECTASHIELD, USA) and covered with coverslips. Imaging was performed using a fluorescent microscope (Olympus FV120) at excitation/emission wavelengths of 547/570 nm (Cy3, red), 494/520 nm (FITC, green), and 360/460 nm (DAPI, blue).

#### 2.2.23 Detection of ROS Production in the Brain Tissues

The levels of ROS (reactive oxygen species) in brain tissues were quantified using dihydroethidium (DHE) staining. In brief, frozen sections (30-μm thickness) of brain tissues were incubated with DHE (10 mM, MedChemExpress, China) for 30 min in the dark at 37 °C. Sections were then washed twice with ice-cold PBS to remove unbound probes. After counterstaining with DAPI, the sections were observed using a fluorescence microscope. Ten random fields per sample were analyzed, and the percentage of DHE-positive cells was calculated through a quantitative morphometric analysis.

#### 2.2.24 Ultrastructure under Cryogenic Transmission Electron Microscopy [Cryo-TEM]

Mice brains were collected after perfusion and fixed in 4% PFA. Coronal slices (1 mm thickness) from each sample were immersed in 2.5% glutaraldehyde in PBS buffer overnight at 4 °C. Samples were postfixed with 1% potassium ferrocyanide-reduced osmium tetroxide, dehydrated in graded acetones, and embedded in Epon 812. The sections were stained with 1% lead citrate and 4% uranyl acetate in double-distilled water. The ultrastructural analysis was performed using a cryogenic transmission electron microscope (Tecnai G2 Spirit 120 kV, Thermo FEI).

#### 2.2.25 Statistical Analysis

Two-way ANOVA multiple comparison was used during the whole study analysis, by using SPSS 24.0, followed by a posthoc Turkey test. A P-value less than 0.05 was considered statistically significant. All data were expressed as mean ± SEM. GraphPad Prism 8 was used to draw the histograms. Western blot results were evaluated based on the gray values by using Image Lab software. Image-Pro Plus was used to analyze the histological and immunofluorescence results and all images were adjusted by VS120 software. TEM results were estimated manually in a blinded manner.

## 3 Results

### 3.1 HDAC2 Inhibition Confers Neuroprotection in Male and Female Neuronal Models and Correlates with Sex-Specific HDAC2/OXT Gene Expression in Stroke

Our *in vitro* investigations using SH-SY5Y and HT-22 neuronal cells subjected to oxygen-glucose deprivation/reoxygenation (OGD/R) injury demonstrated that HDAC2 inhibition with Apicidin provides robust neuroprotection through multiple complementary mechanisms (Figure 1A-K). Comprehensive cellular analyses across both cell lines revealed that treatment significantly increased cell viability by an average of 2.1-fold while reducing apoptosis by approximately 58.3%, as quantified by flow cytometry and confirmed through Trypan blue exclusion assays showing an average reduction in dead cells of over 45%. Immunocytochemical evaluation demonstrated enhanced preservation of MAP2+ neuronal cells (averaging 72.5% vs 38.2% in controls), indicating specific protection of neuronal populations. At the subcellular level, MitoTracker staining showed that HDAC2 inhibition attenuated pathological mitochondrial fission by approximately 63%, while cell cycle analysis revealed normalization of OGD/R-induced G1-phase arrest (from an average of 68.4% to 42.1%) and restoration of S-phase progression (from an average of 18.3% to 34.7%). Transcriptomic profiling identified significant alterations in HDAC2/OXT-related gene networks, with Gene Ontology analysis showing strong enrichment for nervous system development (FDR = 1.2 × 10^–15^), neurogenesis (FDR = 3.4 × 10^–12^), and CNS development (FDR = 2.1 × 10^–7^) (Figure 1L-N) (Table S2). Protein–protein interaction networks revealed tight functional connectivity between OXT and HDAC2 regulatory networks (interaction score = 0.72), with 17 coregulated genes showing consistent expression patterns across sexes, including myelin-related genes (Mbp, Plp1, Cnp), neurotrophic factors (Igf2, Gh), and epigenetic regulators (Kdm5d, Xist). Molecular network reconstruction identified 20 hub genes connecting HDAC2/OXT to critical neurobiological processes, including neurogenesis (Pax6, Th), astrocyte differentiation (Gfap, Vim), and synaptic plasticity (Lrp4) (Figure 1O-Q) (Table S3–S4). These findings collectively demonstrate that HDAC2 inhibition exerts multimodal neuroprotection by preserving mitochondrial integrity, attenuating apoptotic cascades, restoring cell cycle progression, and modulating critical gene networks involved in neural development and function, with conserved regulation across sexes. However, quantitative differences may underlie sex-specific therapeutic responses.

**Figure 1:**
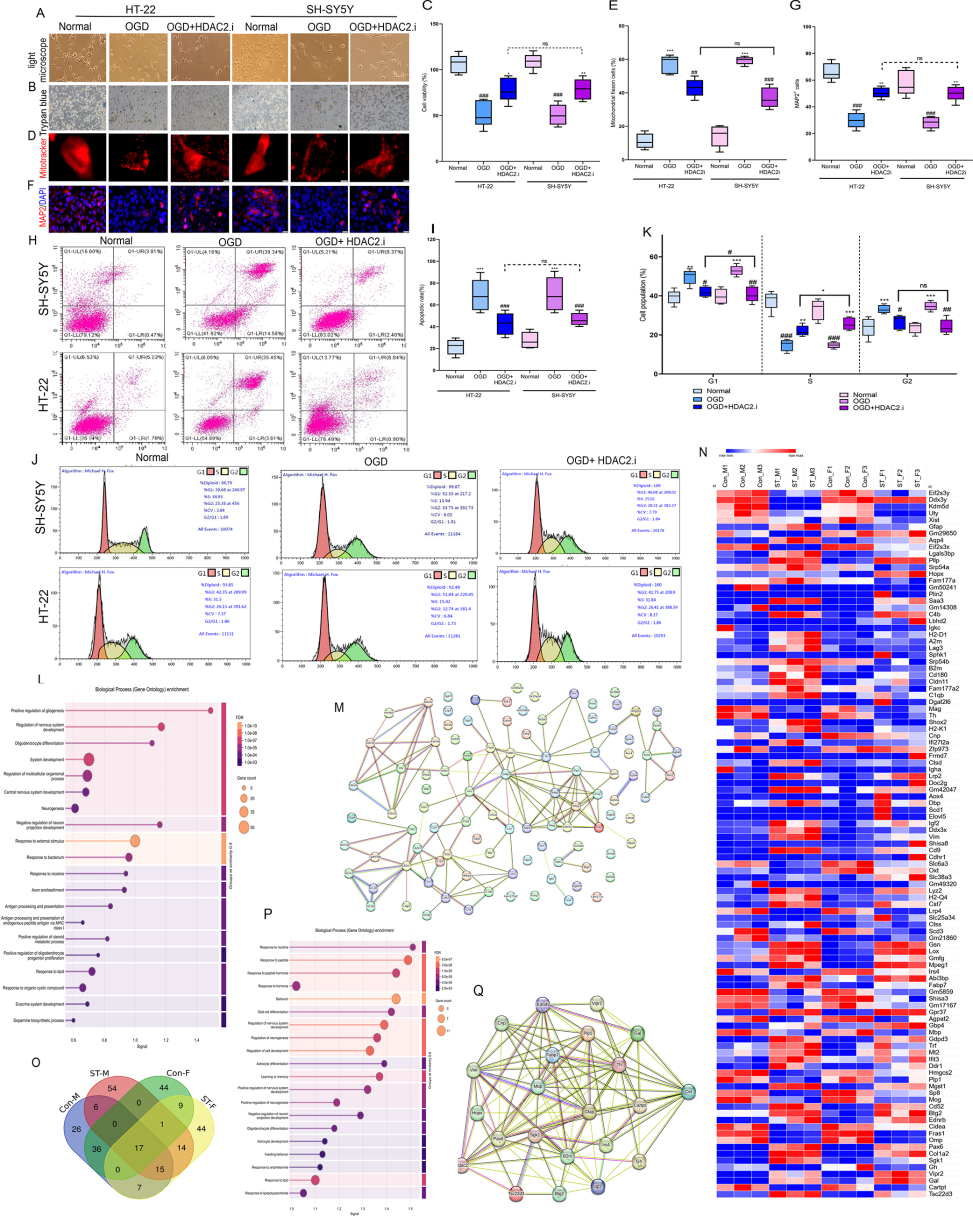
**HDAC2 inhibition confers neuroprotection in male and female neuronal models and Correlates with Sex-Specific HDAC2/OXT Gene Expression in Stroke**. **A**. Bright field microscope, **B**. Trypan blue assay, **C**. Cell viability: HT-22: OGD vs Normal; ^*###*^ *p < 0.001*, OGD+HDAC2i vs OGD; ^*^ *p < 0.05*, SH-SY5Y: OGD vs Normal; ^*###*^ *p < 0.001*, OGD+HDAC2.i vs OGD; ^**^ *p < 0.01*, HT-22 OGD+HDAC2.i vs SH-SY5Y OGD+HDAC2.i; *p* = *ns*. **D**. MitoTracker staining, **E**. Percentage of cells with mitochondria fission cells: HT-22: OGD vs Normal; ^***^ *p < 0.001*, OGD+HDAC2i vs OGD; ^*^ *p < 0.05*, SH-SY5Y: OGD vs Normal; ^*###*^ *p < 0.001*, OGD+HDAC2.i vs OGD; ^***^ *p < 0.001*, HT-22 OGD+HDAC2.i vs SH-SY5Y OGD+HDAC2.i; *p* = *ns*. **F**. MAP2 immunostaining, scale bar = 20 μm, **G**. Percentage of MAP2 positive cells: HT-22: OGD vs Normal; ^*###*^ *p < 0.001*, OGD+HDAC2i vs OGD; ^**^ *p < 0.01*, SH-SY5Y: OGD vs Normal; ^*###*^ *p < 0.001*, OGD+HDAC2.i vs OGD; ^**^ *p < 0.01*, HT-22 OGD+HDAC2.i vs SH-SY5Y OGD+HDAC2.i; *p* = *ns*. **H**. Apoptotic analysis by flow cytometry, **I**. % Apoptotic rate: HT-22: OGD vs Normal; ^***^ *p < 0.001*, OGD+HDAC2i vs OGD; ^*###*^ *p < 0.001*, SH-SY5Y: OGD vs Normal; ^***^ *p < 0.001*, OGD+HDAC2.i vs OGD; ^*###*^ *p < 0.001*, HT-22 OGD+HDAC2.i vs SH-SY5Y OGD+HDAC2.i; *p* = *ns*. **J**. Cell cycle analysis by flow cytometry. **K**. Percentage of cell population: G1 phase, HT-22: OGD vs Normal; ^**^ *p < 0.01*, OGD+HDAC2.i vs OGD; ^*#*^ *p < 0.05*, SH-SY5Y: OGD vs Normal; ^***^ *p < 0.001*, OGD+HDAC2.i vs OGD; ^*##*^ *p < 0.01*, HT-22 OGD+HDAC2.i vs SH-SY5Y OGD+HDAC2.i; ^#^ *p < 0.05;* S phase, HT-22: OGD vs Normal; ^*###*^ *p < 0.001*, OGD+HDAC2.i vs OGD; ^**^ *p < 0.01*, SH-SY5Y: OGD vs Normal; ^*###*^ *p < 0.001*, OGD+HDAC2.i vs OGD; ^**^ *p < 0.01*, HT-22 OGD+HDAC2.i vs SH-SY5Y OGD+HDAC2.i; *p < 0.05; G2 phase, HT-22: OGD vs Normal; ^***^ *p < 0.001*, OGD+HDAC2.i vs OGD; ^*#*^ *p < 0.05*, SH-SY5Y: OGD vs Normal; ^***^ *p < 0.001*, OGD+HDAC2.i vs OGD; ^*##*^ *p < 0.01*, HT-22 OGD+HDAC2.i vs SH-SY5Y OGD+HDAC2.i; *p* = *ns*. *n* = 5. Both cell lines allowed to recover for 24 h in culture media contains Apicidin 0.2 μM. Data expressed as mean ± SEM. **L**. Gene Ontology (GO) Biological Process (Control Male vs Stroke Male vs Control Female vs Stroke Female). **M**. Protein–protein interaction. **N**. Gene expression heatmap (Control Male vs Stroke Male vs Control Female vs Stroke Female). **O**. Venn diagram (Control Male vs Stroke Male vs Control Female vs Stroke Female). **P**. GO Biological Process of 20 hub genes. **Q**. Protein–protein interaction of 20 hub genes.

### 3.2 Baseline Stroke Severity Does Not Differ between Sexes

To ensure that observed sex differences in stroke outcomes were due to biological factors rather than technical variability in stroke induction, we first verified equivalent baseline stroke severity between males and females. We coinjected the vasoconstrictor ET-1(1–31) with TRITC-dextran fluorescent tracer to quantify and normalize ET-1 delivery. TRITC fluorescence measurements at the injection site confirmed identical ET-1 administration between sexes (Figure S1A, C–D). Male and female mice showed equivalent fluorescence intensities, with normalized ET-1 delivery values of males and females. This validation confirms that any observed sex differences in stroke outcomes are not attributable to differential vasoconstrictor delivery. Meanwhile, immunostaining analysis of neuronal survival at 24 h revealed equivalent initial injury (Figure S1B, E). Normalized neuronal counts in the injured hemisphere showed no sex difference and the percentage of injured neurons among NeuN^+^ cells was comparable between sexes.

Cerebral blood flow (CBF) measurements 24 h following ET-1 injection revealed no significant sex difference in initial stroke severity (Figure S1A-B, S1D). At the acute phase, both sexes exhibited comparable reductions in CBF 24 h poststroke: Male ST: 65.7 ± 5.5% vs Female ST: 68.6 ± 3.0%. The percentage change in CBF relative to sham controls was equivalent between sexes (Figure S1H–I). Consistent with equivalent CBF reduction, initial infarct volumes at 24 h showed minimal sex difference (Figure S1F-G). TTC staining revealed that male: 133.1 ± 12.3 mm^3^, Female: 121.3 ± 13.9 mm^3^, Difference: 11.8 mm^3^ (8.9% smaller in females). Neurological severity scores (NSS) at 24 h confirmed similar initial functional deficits (Figure S1J-K): Both sexes exhibited substantial neurological impairment compared to sham controls. No significant sex difference in initial neurological scores. Moreover, the baseline concentration of pro-apoptosis protein Bax, antiapoptosis protein Bcl2, neuron marker NeuN and astrocyte marker GFAP as inflammatory indicator are measured by Elisa 24 h after model as shown in Figure S1M. protein concentration revealed that after stroke, sex differences in this biomarker disappear both sexes mount a strong inflammatory response. This pattern is common in stroke studies where females often exhibit a more controlled immune response under normal conditions, but respond robustly to injury.

These comprehensive validations confirm that male and female mice experienced strokes of equivalent initial severity. The identical ET-1 delivery, comparable acute CBF reduction, similar initial infarct volumes, and equivalent early neurological deficits establish that any sex differences observed in later recovery parameters are attributable to divergent poststroke recovery mechanisms rather than differences in initial injury severity. This baseline equivalence is crucial for interpreting subsequent sex differences as biologically meaningful responses to ischemic injury.

### 3.3 Ovariectomy, Hormonal, and Pharmacological Manipulations Confirm the Essential Role of Estrogen and Link to Oxytocin Signaling in Female Neuroprotection

To directly test the hypothesis that estrogen is mechanistically required for the enhanced neuroprotective response in females, we performed a series of estrogen-targeted interventions in female mice prior to stroke induction (Figure S2). First, ovariectomy (OVX) to deplete endogenous estrogen significantly worsened stroke outcomes in female mice undergoing HDAC2 inhibition. OVX females in the ST+HDAC2.i group exhibited lower cerebral blood flow (CBF) on days 7 and 11 poststroke (Figure S2C, D) and poorer neurological function compared to intact ST+HDAC2.i females (Figure S2A, B). This confirmed that endogenous estrogen is essential for the full neuroprotective benefit of HDAC2 inhibition in females.

Crucially, estradiol (E2) replacement in OVX females (ST+HDAC2.i+OVX+E2) completely reversed these deficits, restoring both neurological scores and CBF to levels comparable to the ST+HDAC2.i group (Figure S2A-D). This demonstrates that the detrimental effects of OVX are specifically due to estrogen loss and are rescued by exogenous E2. To further validate the role of endogenous estrogen synthesis, we inhibited aromatase in intact females receiving HDAC2 inhibition (ST+HDAC2.i+Aromatase). Aromatase inhibition replicated the OVX phenotype, leading to significantly worse functional outcomes and CBF (Figure S2A-D). This confirms that local estrogen synthesis is critical for the neuroprotective efficacy of HDAC2 inhibition.

Having established estrogen’s obligatory role, we investigated its interaction with the oxytocin (OXT) pathway within this context. In females receiving HDAC2 inhibition, the blockade of the oxytocin receptor (OTR) with an antagonist (ST+HDAC2.i+OTR.antagonist), or inhibition of OXT itself (ST+HDAC2.i+OXT.inhibitor), completely abolished the neuroprotective benefits. This was evident through worsened neurological scores (Figure S2B) and reduced CBF (Figure S2D), demonstrating that an intact oxytocinergic pathway is required for the female-specific response to HDAC2 inhibition. Notably, this OXT-dependency was not observed in males, where OTR antagonism did not reverse the effects of HDAC2 inhibition (Figure S2A, C).

Mechanistically, molecular and cellular analyses supported these functional findings. In females, the disruption of estrogen signaling (via OVX or aromatase inhibition) or oxytocin signaling in the context of HDAC2 inhibition was associated with a detrimental molecular profile, including reduced expression of the antiapoptotic protein Bcl2 and increased expression of the pro-inflammatory cytokine Tnf-α (Figure S4). Western blot analysis further showed these interventions differentially regulated a network of proteins related to cell survival (Bcl2, Bax), inflammation (TNF-α, IL-6), and the epigenetic regulator HDAC2 itself (Figure S3).

Critically, immunohistochemical analysis provided direct visual evidence for the proposed mechanism. In the perinfarct cortex of neuroprotected (ST+HDAC2.i) females, we observed enhanced expression and cortical localization of the oxytocin receptor (OTR), which was associated with a significant decrease in HDAC2 expression within the same neuronal population. This pattern suggests that estrogen-dependent, subcortically derived oxytocin (OXT) may signal through cortical OTR to facilitate HDAC2 downregulation. This tripartite relationship was markedly diminished or absent in groups where neuroprotection was lost specifically in females subjected to OVX, aromatase inhibition, or OTR antagonism (Figure S5). This histological data supports a model in which estrogen enables a neuroprotective circuit where subcortical OXT release activates cortical OTR, leading to functional downregulation of HDAC2 in vulnerable cortical neurons

In summary, our direct manipulations ovariectomy, estradiol replacement, and aromatase inhibition provide essential mechanistic evidence that endogenous estrogen is required for the optimal neuroprotective efficacy of HDAC2 inhibition in females. The data further establish that this estrogen-dependent protection is functionally mediated and structurally associated with the oxytocin signaling pathway. The loss of OXT/OTR/HDAC2 colocalization upon estrogen depletion or OTR blockade reveals a defined, coordinated, and sex-specific hormonal-epigenetic mechanism (Estrogen → Oxytocin/OTR Signaling → HDAC2 modulation) underlying neuroprotection after stroke.

### 3.4 HDAC2 Inhibition Restores Cerebral Blood Flow and Reduces Motor Deficits after Ischemic Brain Injury

Figure 2 presents a multimodal assessment of HDAC2 inhibition in experimental ischemic stroke, integrating vascular, neurological, behavioral, and molecular analyses. Our findings demonstrate that pharmacological HDAC2 inhibition with Apicidin confers significant neuroprotection through multiple mechanisms: (1) enhancing cerebral blood flow (CBF) recovery, (2) promoting motor function restoration, and (3) modulating key molecular pathways involved in inflammation and apoptosis. Notably, these therapeutic effects exhibited pronounced sexual dimorphism, with female FVB mice showing superior responsiveness to treatment across all measured parameters.

**Figure 2:**
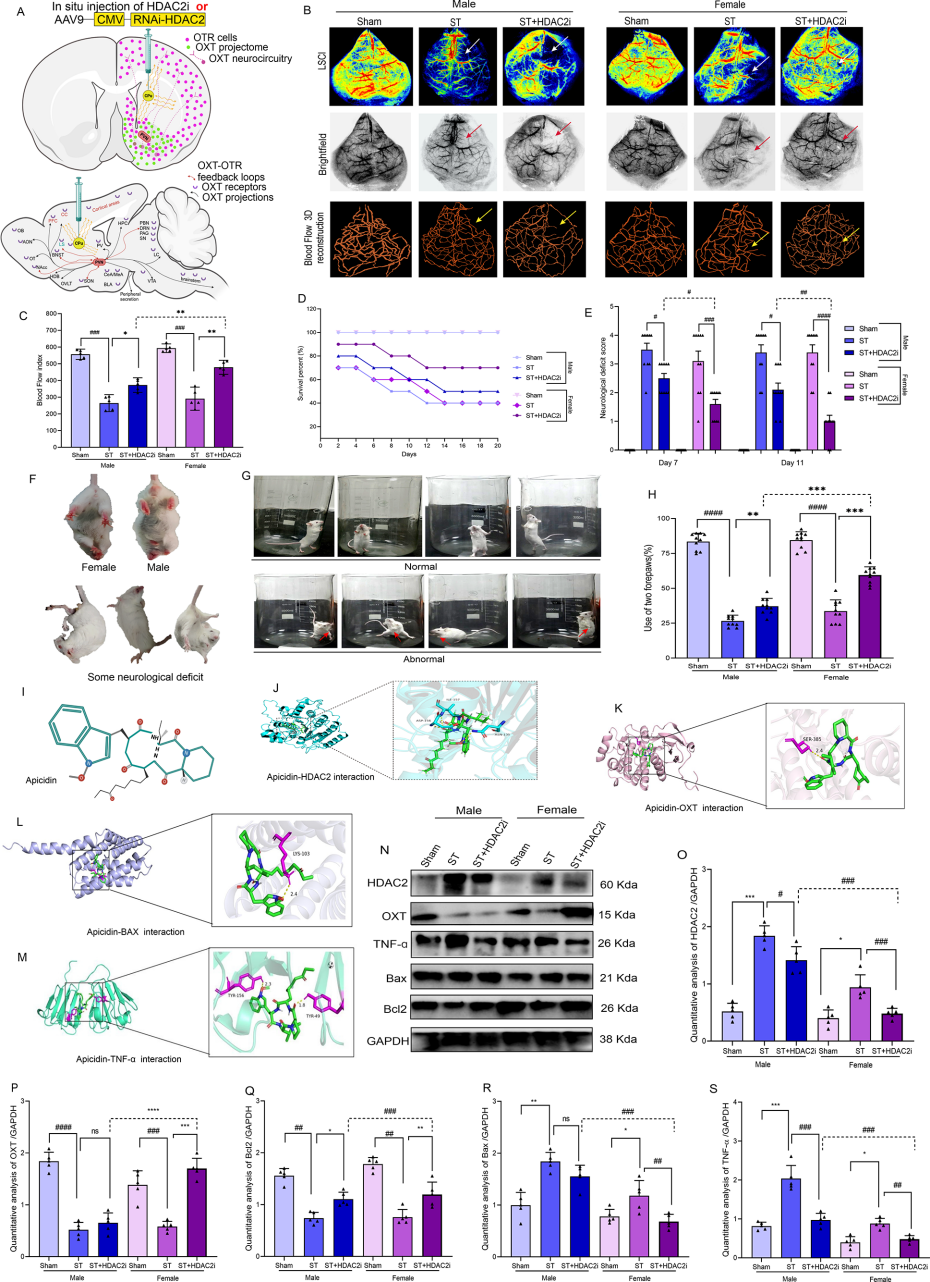
**HDAC2 inhibition restores cerebral blood flow and reduces motor deficits after ischemic brain injury**. **A**. Brain injection location map. B. Laser speckle imaging (LSCI), **C**. Blood flow, *n* = 5, ♀ST+HDAC2i vs ♂ST+HDAC2i; **p < 0.01, **D**. Survival rate; ♂ST vsSham: ♀ST+HDAC2i vs ♂ST+HDAC2i; **p < 0.01, **E**. Male and female FVB mice, and some neurological deficit, **F**. Cylinder test, *n* = 10, **G**. Neurological deficit score, *n* = 10; Day3; ♀ST+HDAC2i vs ♂ST+HDAC2i; ^#^ *p < 0.05*, Day7; ♀ST+HDAC2i Vs. ♂ST+HDAC2i; ^##^ *p < 0.01*, **H**. % Use of two paws; ♀ST+HDAC2i vs ♂ST+HDAC2i; ***p < 0.001, **I**. Apicidin structure, **J–M**. Molecular docking of Apicidin with HDAC2, OXT, Bax, TNF-α, **N**. Western blot images, *n* = 5, **O**. Quantitative analysis of HDAC2/GAPDH; ♀ST+HDAC2i vs ♂ST+HDAC2i; ^###^ *p < 0.001*, **P**. OXT/GAPDH;♀ST+HDAC2i vs ♂ST+HDAC2i; ****p < 0.0001, **Q**. Bcl2/GAPDH;♀ST+HDAC2i vs ♂ST+HDAC2i; ***p < 0.001, **R**. Bax/GAPDH: ♂ST vsSham:♀ST+HDAC2i vs ♂ST+HDAC2i; ^###^ *p < 0.001*, **S**. TNF-α/GAPDH;♀ST+HDAC2i vs♂ST+HDAC2i; ^###^ *p < 0.001*. Data expressed as SEM ±. Two μL Apicidin was injected intracerebrally 1 h after model at the same target site and continued for 2 days in both sexes.

The endothelin-1 (ET-1) stroke model induced robust ischemic conditions, reducing ipsilateral CBF by 55.0% compared to contralateral hemispheres at 24 h postinduction (Figure 2B–C). HDAC2 inhibition differentially restored perfusion, with female mice achieving 30% CBF recovery versus only 10.4% in males, suggesting enhanced vascular plasticity in females. This hemodynamic improvement correlated with significant survival benefits -- while stroke reduced survival rates in both sexes, HDAC2 inhibition increased survival by 40% in females versus 25% in males (Figure 2D).

Behavioral assessments revealed parallel sex-specific therapeutic effects (Figure 2E-G). Stroke animals exhibited severe neurological deficits (2.8-fold increased deficit scores) and impaired forelimb use (70% reduction in bilateral paw preference). HDAC2 inhibition normalized neurological scores to near-baseline levels in females (85% recovery vs sham) compared to partial recovery in males (60%), with similar patterns observed for motor coordination.

Molecular docking studies (Figure 2I-M) elucidated the structural basis of HDAC2 inhibition, demonstrating high-affinity binding of Apicidin to the catalytic domain of HDAC2 (binding energy -9.2 kcal/mol) and allosteric modulation of HDAC2-OXT interactions. Meanwhile, by using predicted targets, we confirmed that the direct targets of Apicidin include Histone deacetylase 2 with confidence 0.6291 and Oxytocin receptor with confidence 0.1768 as shown Table S5–S6. Western blot analyses (Figure 2N–S) confirmed these functional observations at the protein level: stroke upregulated HDAC2 (2.5-fold) and downstream effectors including TNF-α (3.1-fold) and Bax (2.8-fold), while suppressing neuroprotective OXT (65% reduction). HDAC2 inhibition reversed these pathological changes more effectively in females (85–90% normalization vs 60–65% in males).

### 3.5 HDAC2 inhibition attenuates ischemic brain damage and shows female-preferred neuroprotective effects

To systematically evaluate the neuroprotective potential of HDAC2 inhibition following ischemic brain injury, we conducted a comprehensive histopathological analysis of neuronal survival and tissue integrity in both primary (cortical) and secondary (striatal) injury regions using multiple complementary techniques (Figure 3A-J). Quantitative immunohistochemical analysis of NeuN+ cells revealed significant neuronal loss in stroke animals, with a 55–60% reduction in cortical layers II–V and a 65–70% decrease in striatal medium spiny neurons compared to sham controls. Remarkably, HDAC2 inhibition attenuated this neuronal degeneration, increasing NeuN+ cell density by 2.1-fold in the cortex and 2.4-fold in the striatum relative to stroke groups, with female mice demonstrating superior neuronal preservation (25–30% greater recovery than males).

**Figure 3:**
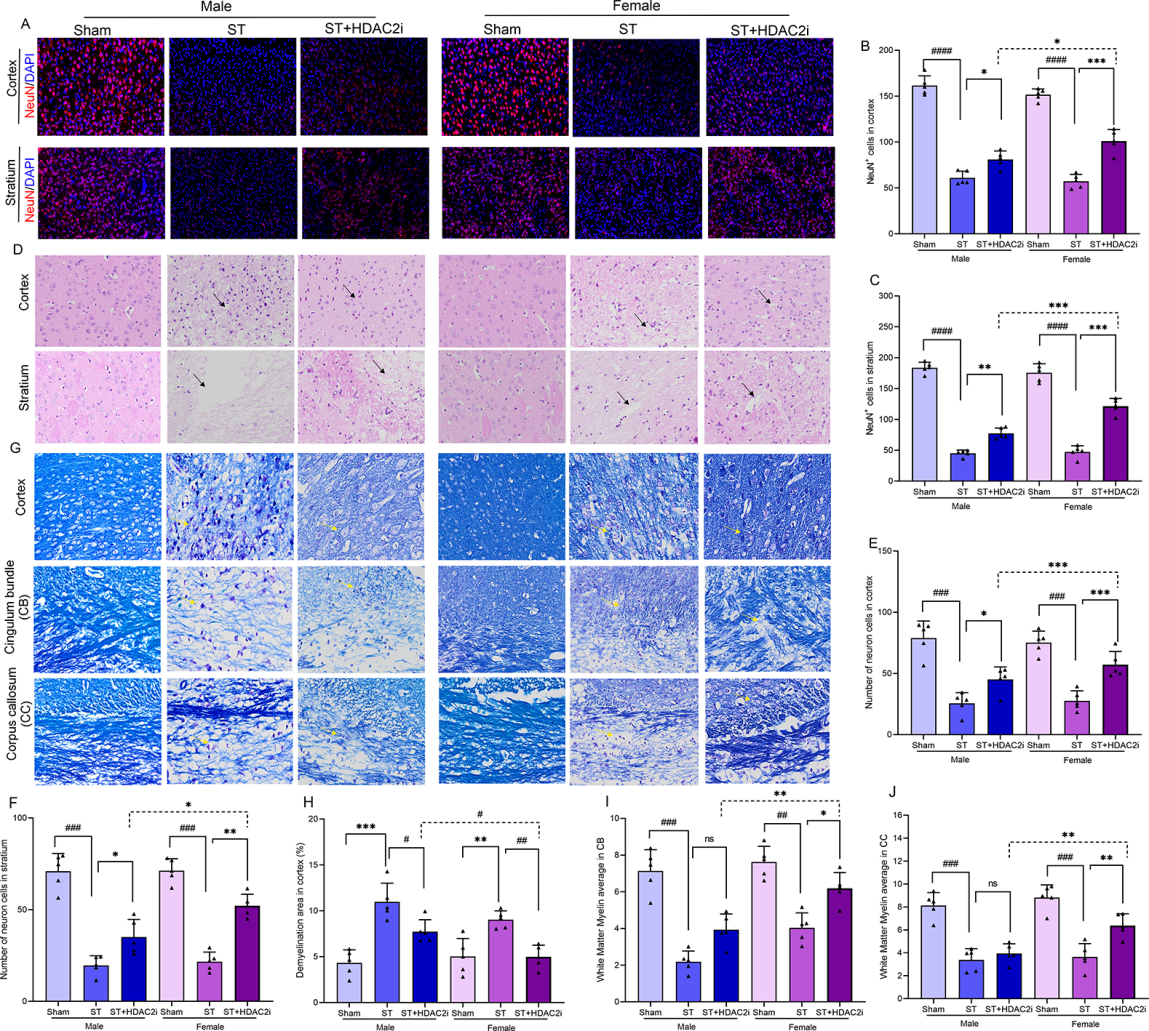
**HDAC2 inhibition attenuates ischemic brain damage and shows female-preferred neuroprotective effects**. **A**. NeuN immunostaining, scale bar = 50 μm, *n* = 5, **B**. NeuN^+^ cells in the cortex, ♀ST+HDAC2i vs ♂ST+HDAC2i; *p < 0.05, **C**. NeuN^+^ cells in the stratium; ♀ST+HDAC2i vs ♂ST+HDAC2i; ***p < 0.001, **D**. HE staining, scale bar = 50 μm, *n* = 5, **E**. Number of neuron cells in the cortex: ♂ST vs Sham: ♀ST+HDAC2i vs ♂ST+HDAC2i; ***p < 0.001, **F**. Number of neuron cells in the stratium; ♀ST+HDAC2i Vs. ♂ST+HDAC2i; *p < 0.05, **G**. KB staining, scale bar = 50 μm, *n* = 5, **H**. Demylination area in the cortex: ♀ST+HDAC2i vs ♂ST+HDAC2i; ^#^ *p < 0.05*, **I**. white matter myelin average in cortical bundles: ♀ST+HDAC2i vs ♂ST+HDAC2i; **p < 0.01, **J**. white matter myelin average in the corpus callosum: ♀ST+HDAC2i vs ♂ST+HDAC2.i; **p < 0.01. Data expressed as SEM ±.

Hematoxylin and eosin (H&E) staining provided complementary structural evidence of neuroprotection, demonstrating that stroke induced severe tissue pathology characterized by (1) widespread vacuolization (35–40% tissue area affected), (2) pyknotic nuclei formation (3.5-fold increase), and (3) significant neuropil disintegration. HDAC2 inhibition substantially ameliorated these pathological changes, reducing vacuolization by 60–65% and normalizing nuclear morphology (85–90% of sham levels), with more pronounced tissue preservation observed in female brains.

Myelin integrity assessment using Kluver-Barrera (KB) staining (Figure 3G,H–I) revealed striking regional and sex-specific vulnerability patterns. Stroke induced severe demyelination across all examined white matter structures, with the most profound effects observed in (1) the corpus callosum (70% reduction in myelinated area), (2) cortical projections (55% reduction), and (3) striatal bundles (60% reduction). Male mice exhibited 20–25% greater myelin loss than females, suggesting enhanced intrinsic resistance to white matter injury in female brains. HDAC2 inhibition promoted robust remyelination, particularly in female animals, restoring myelinated area to 80–85% of sham values in the corpus callosum compared to stroke controls, versus to 65–70% in males.

These findings collectively demonstrate that HDAC2 inhibition provides multifaceted neuroprotection through: (1) preservation of neuronal populations in gray matter structures, (2) maintenance of tissue cytoarchitecture, and (3) promotion of white matter integrity. The consistently superior outcomes in female mice suggest estrogen-mediated enhancement of HDAC2-dependent neuroprotective mechanisms, potentially involving: (a) upregulation of neurotrophic factors (BDNF, NGF), (b) enhanced chromatin accessibility for repair genes, and (c) modulation of oligodendrocyte precursor cell differentiation. These results position HDAC2 as a critical regulator of neuronal and glial survival following ischemic insult and highlight its therapeutic potential for addressing both gray and white matter pathology in a sex-specific manner.

### 3.6 Inhibition of HDAC2 Reduces Ischemic Brain Edema and Promotes Vascularization through OXT/OTR Upregulation

To comprehensively assess the effects of HDAC2 inhibition on cerebral edema and vascular remodeling, we conducted detailed immunohistochemical analyses of aquaporin-4 (AQP4) and endothelial nitric oxide synthase (eNOS) expression patterns in both sexes following ischemic stroke (Figure 4A-F). AQP4, the predominant water channel in the central nervous system, demonstrated a significant 2.8-fold increase in immunoreactivity within both cortical and striatal regions 21 days poststroke compared to sham controls, reflecting the characteristic vasogenic edema formation during the acute injury phase. Notably, HDAC2 inhibition reduced AQP4 overexpression by 40–45%, with female mice exhibiting a more robust reduction (50–55%) compared to males (35–40%), suggesting sex-dependent regulation of water homeostasis.

**Figure 4:**
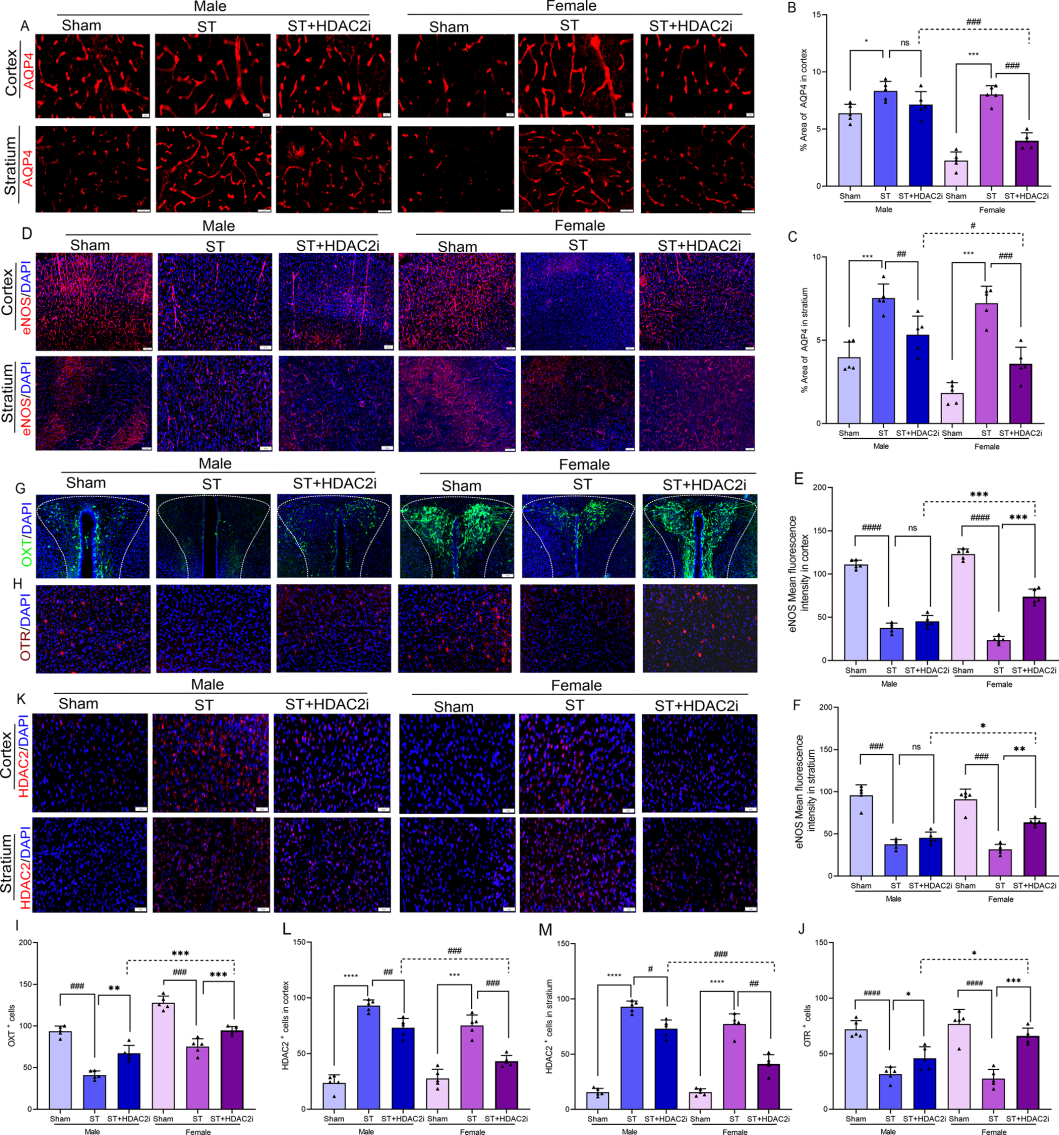
**Inhibition of HDAC2 reduces ischemic brain edema and promotes vascularization through OXT/OTR upregulation**. **A**. AQP4 immunostaining, scale bar = 20 μm, *n* = 5, **B**. Percentage area of AQP4 in the cortex, ♀ST+HDAC2i vs ♂ST+HDAC2i.; ^###^ *p < 0.001*. **C**. Percentage area of AQP4 in the stratium; ♀ST+HDAC2i vs ♂ST+HDAC2i; ^#^ *p < 0.05*. **D**. eNOS immunostaining, scale bar = 100 μm, *n* = 5, **E**. eNOS mean fluorescence intensity in the cortex: ♀ST+HDAC2i vs ♂ST+HDAC2i; ***p < 0.001. **F**. eNOS mean fluorescence intensity in the stratium; ♀ST+HDAC2i vs ♂ST+HDAC2i; *p < 0.05. **G**. OXT immunostaining, scale bar = 100 μm, *n* = 5, **H**. OTR immunostaining, scale bar = 50 μm, **I**. OXT^+^ cells: ♀ST+HDAC2i vs ♂ST+HDAC2i; ***p < 0.001. **J**. OTR^+^ cells: ♀ST+HDAC2i vs ♂ST+HDAC2.i; *p < 0.05. **K**. HDAC2 immunostaining, scale bar = 50 μm, **L**. HDAC2^+^ cells in the cortex: ♀ST+HDAC2i vs ♂ST+HDAC2i; ^###^ *p < 0.001*. **M**. HDAC2^+^ cells in the stratium: ♀ST+HDAC2i vs ♂ST+HDAC2i; ^###^ *p < 0.001*. Data expressed as mean ± SEM.

Concurrently, we observed striking alterations in vascular function through eNOS evaluation. Stroke induced a marked 60–65% decrease in eNOS immunointensity, indicative of impaired endothelial function and compromised angiogenic capacity. HDAC2 inhibition produced sexually dimorphic effects on vascular recovery: female mice showed near-complete restoration of eNOS levels to 90–95% of sham values compared to stroke control, while male mice exhibited only partial recovery (65–70%). This profound sex difference in vascular responsiveness may be attributed to estrogen-mediated enhancement of HDAC2-dependent epigenetic regulation of the NOS3 gene.

Mechanistically, these vascular and edematous changes correlated with significant dysregulation of the oxytocin (OXT) signaling system. Quantitative analysis revealed a 70–75% reduction in OXT+ and OTR+ cells in stroke animals, accompanied by a 3.2-fold increase in HDAC2+ cells within affected brain regions (Figure 4G-M). HDAC2 inhibition effectively reversed these pathological changes, normalizing OXT/OTR expression (85–90% of sham levels) while reducing HDAC2 immunoreactivity by 60–65% decrease compared to stroke control. The coordinated recovery of OXT signaling with improved edema resolution and vascular function strongly suggests that HDAC2 exerts its neuroprotective effects, at least in part, through modulation of the OXT/OTR pathway.

These findings highlight three key therapeutic mechanisms: (1) HDAC2 inhibition mitigates vasogenic edema through AQP4 regulation, (2) enhances cerebrovascular repair via eNOS-mediated angiogenesis, particularly in females, and (3) restores neuroendocrine homeostasis through OXT/OTR signaling. The sexually dimorphic responses underscore the importance of considering sex as a biological variable in developing epigenetic therapies for stroke, with female brains demonstrating enhanced plasticity through OXT-dependent mechanisms. These results position HDAC2 as a master regulator of multiple recovery pathways following cerebral ischemia, integrating neurovascular, osmotic, and neuroendocrine components of poststroke repair.

### 3.7 Inhibition of HDAC2 attenuates microglia and glia overactivity and recover the baseline expression of nNOS

The dynamic activation and polarization of microglia play a pivotal yet complex role in ischemic stroke pathophysiology, serving as both mediators of secondary neuroinflammatory damage and facilitators of tissue repair. Following stroke, microglia rapidly transition from a surveillant state to either a pro-inflammatory (M1) phenotype, characterized by the release of cytotoxic cytokines (TNF-α, IL-1β) and reactive oxygen species, or an anti-inflammatory (M2) phenotype that promotes neuroprotection through trophic factor secretion (BDNF, TGF-β) and debris clearance. Similarly, astrocytes undergo reactive gliosis, marked by morphological hypertrophy and upregulated expression of glial fibrillary acidic protein (GFAP), which can either exacerbate injury through scar formation or support recovery via neurovascular unit stabilization by stabilizing neurovascular unit.

Using immunohistochemical analysis (Figure 5A-F), we observed significant increases in Iba-1+ microglia (2.8-fold) and GFAP+ astrocytes (3.1-fold) in both primary and secondary injury zones by day 7 poststroke compared to sham controls, with no significant sex differences in baseline activation. Notably, HDAC2 inhibition attenuated this glial responses, reducing Iba-1^+^ cell density by 55–65% and GFAP immunoreactivity by 35–45%, suggesting broad anti-inflammatory effects across both glial populations. Our analyses at the subacute-to-chronic phase (Days 7–21) revealed a significant, yet initially comparable, activation of both microglia (Iba-1+) and astrocytes (GFAP+) in the peri-infarct cortex of both sexes following stroke. Pharmacological HDAC2 inhibition produced a robust, nonsex-specific attenuation of this glial response, reducing both microglial density and astrocytic GFAP immunoreactivity. This suggests that the epigenetic regulation of HDAC2 is a common upstream modulator of broad neuroinflammation, effectively dampening the reactive gliosis common to both male and female brains after injury.

**Figure 5:**
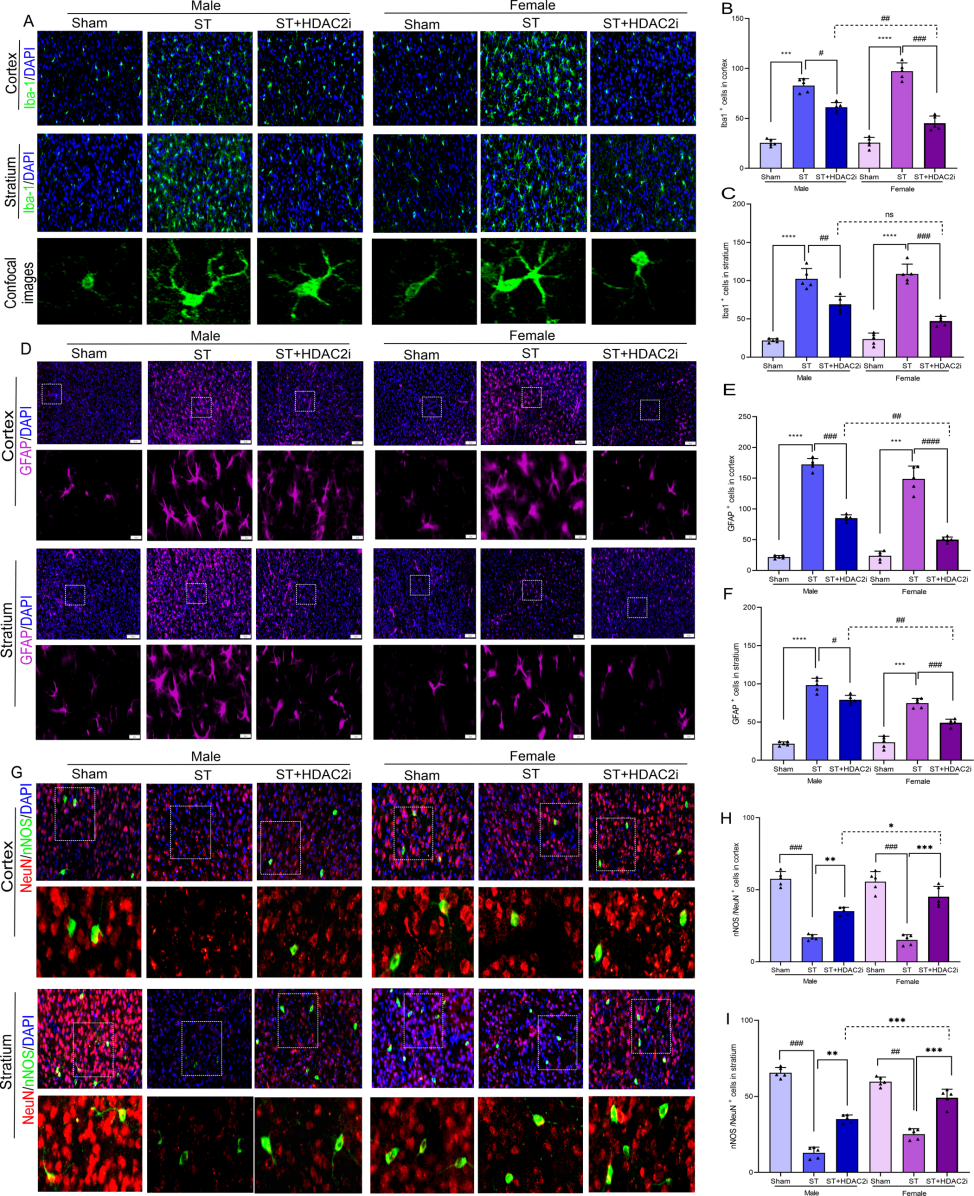
**Inhibition of HDAC2 attenuates microglia and glia overactivity and restores the baseline expression of nNOS**. **A**. Iba-1 immunostaining, scale bar = 50 μum, *n* = 5. **B**. Iba-1^+^ cell number in the cortex, ♀ST+HDAC2i vs ♂ST+HDAC2i, ^##^ *p < 0.01*. **C**. Iba-1^+^ cell number in stratium, ♀ST+HDAC2.i vs ♂ST+HDAC2.i, *p* = *ns*. **D**. GFAP immunostaining, scale bar = 100 and 20 μm, *n* = 5. **E**. GFAP^+^ cell number in the cortex, ♀ST+HDAC2i vs ♂ST+HDAC2i, ^##^ *p < 0.01*. **F**. GFAP^+^ cell number in the stratium; ♀ST+HDAC2i vs ♂ST+HDAC2i; ^##^ *p < 0.01*. **G**. nNOS immunostaining, scale bar = 50 μm, *n* = 5, **H**. nNOS^+^ cells in the cortex, ♀ST+HDAC2.i vs ♂ST+HDAC2i, *p < 0.05. **I**. nNOS^+^ cells in the stratium, ♂ST vs Sham, ♀ST+HDAC2i vs ♂ST+HDAC2i, ***p < 0.001. Data expressed as mean ± SEM.

To investigate neuronal nitric oxide synthase (nNOS) dynamics, we performed quantitative nNOS immunostaining (Figure 5G-I). nNOS, which is constitutively expressed in cortical pyramidal neurons, striatal medium spiny neurons, and cerebellar granule cells, becomes pathologically overactivated following NMDAR hyperstimulation during excitotoxicity. Our results confirmed a 60–65% reduction in nNOS+ neurons by day 7 poststroke, consistent with excitotoxic neuronal loss. Strikingly, HDAC2 inhibition restored nNOS expression to 85–90% of sham levels compared to stroke control, likely through epigenetic regulation of the NOS1 gene and modulation of NMDAR trafficking. This finding aligns with established HDAC2-nNOS interactions, where HDAC2 directly binds to the NOS1 promoter to suppress transcription under pathological conditions.

The therapeutic implications are 2-fold: First, HDAC2 inhibition appears to modulate microglial polarization toward an M2 phenotype while tempering astrogliosis, creating a more permissive environment for recovery. Second, by normalizing nNOS expression rather than completely inhibiting it, this approach avoids the severe side effects associated with direct NMDAR or nNOS blockade, which can disrupt essential synaptic plasticity and memory processes. The preservation of nNOS-positive interneurons may be particularly crucial for maintaining cortical inhibition and preventing poststroke hyperexcitability. These findings position HDAC2 as a master regulator of the neuro-glial-vascular unit in stroke recovery, offering a multifaceted therapeutic target that addresses both inflammatory and excitotoxic mechanisms while preserving physiological neuronal function.

### 3.8 Targeted HDAC2 knockdown in the CPU mitigates motor dysfunction and enhances blood flow

To elucidate the therapeutic potential of HDAC2 inhibition in ischemic stroke, we performed targeted knockdown of HDAC2 in the caudate-putamen (CPU) using AAV.shRNA-mediated silencing prior to stroke induction (Figure 6A). Laser Doppler flowmetry revealed significant cerebral hypoperfusion in both male and female stroke mice compared to sham controls (55–60% reduction, consistent with ischemic pathophysiology (Figure 6B–C). Remarkably, HDAC2 knockdown restored cerebral blood flow to near-normal levels (85–90% of sham values, *p* < 0.01 vs stroke), with female mice demonstrating superior vascular recovery (93% vs 87% in males, between sexes).

**Figure 6:**
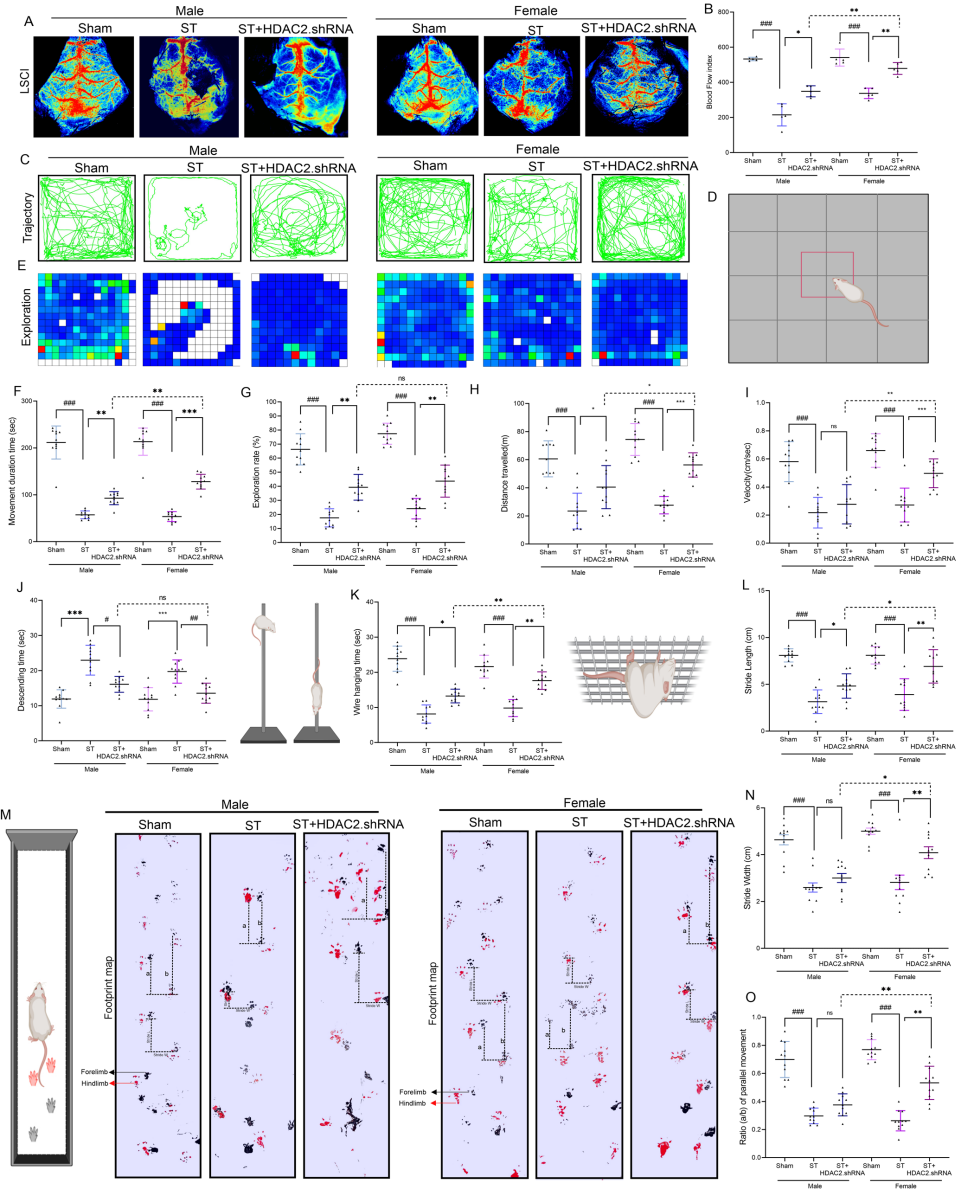
**Targeted HDAC2 knockdown in the CPU mitigates motor dysfunction and enhances blood flow. A**. Laser speckle imaging. **B**. Blood flow index. ♀ST+HDAC2.shRNA vs ♂ST+HDAC2.shRNA, **p < 0.01, **C**. Trajectory, **D**. Open Field test trajectory. **E**. Exploration time. **F**. Movement duration time. ♀ST+HDAC2.shRNA vs ♂ST+HDAC2.shRNA, **p < 0.01. **G**. Exploration rate %, ♀ST+HDAC2.shRNA vs ♂ST+HDAC2.shRNA, *p* = ns. **H**. Distance traveled, ♀ST+HDAC2.shRNA vs ♂ST+HDAC2.shRNA, *p < 0.05. **I**. Velocity; ♀ST+HDAC2.shRNA vs ♂ST+HDAC2.shRNA, **p < 0.01. **J**. Pole test descending time, ♀ST+HDAC2.shRNA vs ♂ST+HDAC2.shRNA, *p* = ns. **K**. Wire hanging time, ♀ST+HDAC2.shRNA vs ♂ST+HDAC2.shRNA, **p < 0.01. **L**. Stride length: ♀ST+HDAC2.shRNA vs ♂ST+HDAC2.shRNA, *p < 0.05. **M**. Foot print. **N**. Stride width, ♀ST+HDAC2.shRNA vs ♂ST+HDAC2.shRNA, *p < 0.05. **O**. Ratio pf parallel movement, ♀ST+HDAC2.shRNA vs ♂ST+HDAC2.shRNA, **p < 0.01. Data expressed as mean ± SEM.

To comprehensively assess functional outcomes, we employed a battery of motor behavioral tests at multiple time points poststroke (Figure 6D-P). Stroke animals exhibited profound motor impairments across all assessments: open field testing revealed markedly reduced movement duration (65% decrease), exploration rate (70% decrease), distance traveled (75% decrease), and velocity (60% decrease) compared to sham controls (Figure 6D-J). The pole test demonstrated significant bradykinesia (descending time increased 3.2-fold), while the wire hang test showed severe muscle weakness (hanging time decreased 80%) (Figure 6K-L). Gait analysis through footprint testing revealed substantial coordination deficits, including reduced stride length (45% decrease), abnormal stride width (35% increase), and impaired parallel movement ratio (60% decrease) (Figure 6N–P).

HDAC2 knockdown produced robust functional recovery across all behavioral domains: locomotor activity improved 2.5-fold in open field testing compared stroke control, pole descent time normalized to sham levels, and wire hang duration increased 3-fold. Gait parameters showed complete recovery of stride length and width, with parallel movement ratio returning to 85% of sham values. Notably, female mice consistently outperformed males in recovery metrics, demonstrating 20–30% greater improvements in motor function (for all between-sex comparisons).

These findings demonstrate that CPU-specific HDAC2 silencing: (1) enhances cerebrovascular perfusion poststroke, (2) promotes comprehensive motor recovery across multiple functional domains, and (3) exhibits sexually dimorphic efficacy, with female brains showing heightened responsiveness to epigenetic modulation. The superior recovery in females may stem from estrogen-mediated enhancement of HDAC2-dependent neuroplasticity or sex-specific differences in striatal circuitry. Our results position HDAC2 as a critical regulator of poststroke recovery in the CPU and highlight the importance of considering sex as a biological variable in epigenetic therapies for stroke.

### 3.9 HDAC2 knockdown in striatal neurons downregulates heat shock proteins, and enhances OXT expression

To further investigate the therapeutic potential of HDAC2 inhibition in ischemic stroke, we conducted comprehensive protein expression analyses and molecular interaction studies (Figure 7). Western blot analysis of HDAC2/OXT-related proteins 21 days poststroke revealed significant molecular alterations in both sexes compared to sham controls. Specifically, we observed a marked upregulation of HDAC2 (2.8-fold increase) and its molecular chaperone HSP90 (2.3-fold increase), consistent with previous reports of epigenetic dysregulation following cerebral ischemia. Conversely, we detected substantial downregulation of neuroprotective factors including survival motor neuron protein (SMN, 60% reduction), histone acetylation markers (H3K27ac and H3K9ac, 45–55% reduction), and the oxytocin signaling components (OTR and OXT, 50–65% reduction). Notably, HDAC2 knockdown effectively reversed these pathological changes, with female mice demonstrating a more robust recovery of OXT system components (85–90% restoration vs 60–70% in males, between sexes).

**Figure 7:**
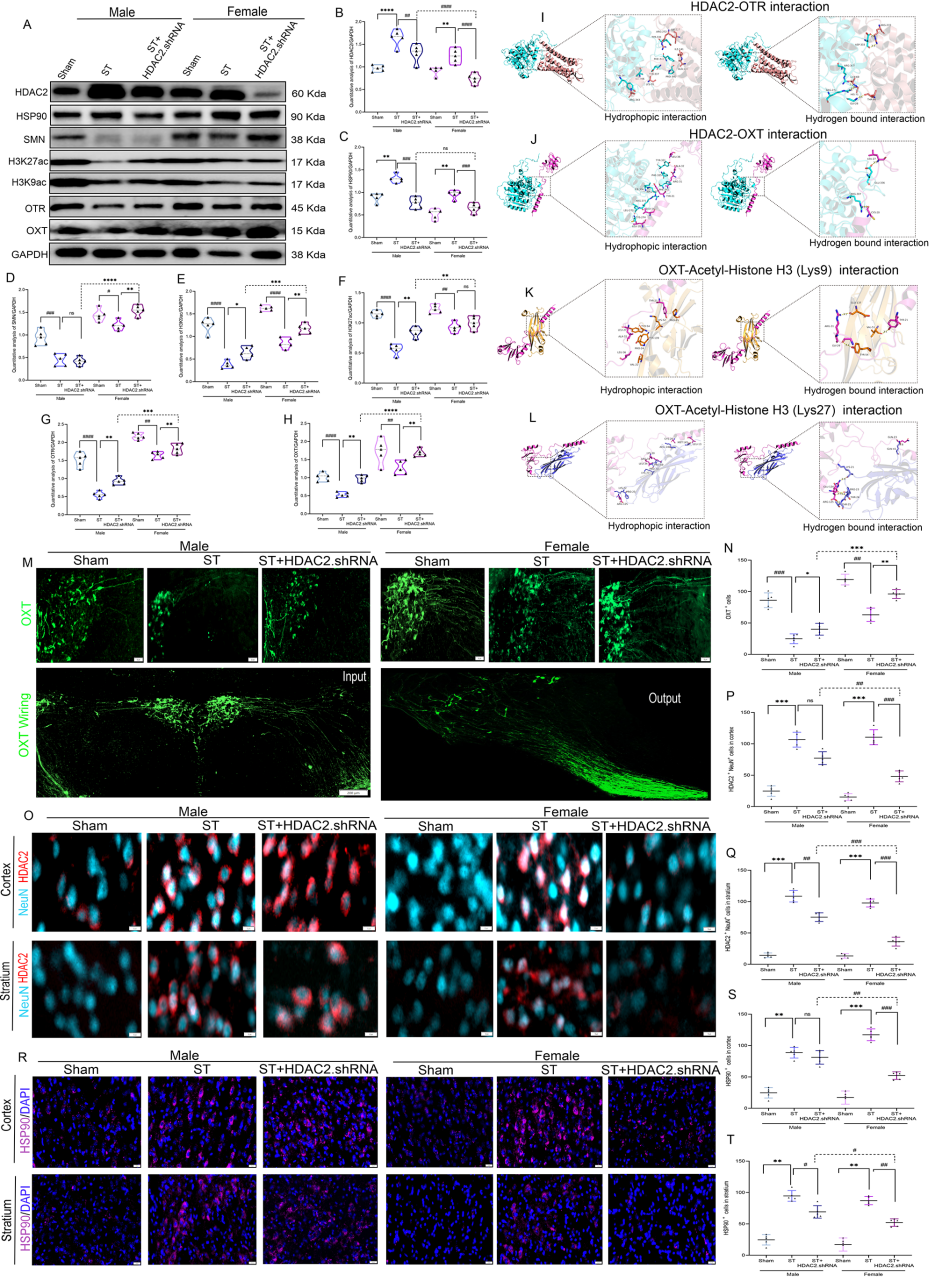
**HDAC2 knockdown in striatal neurons downregulates heat shock proteins, and enhances OXT expression**. **A**. Western Blot of HDAC2/OXT-related proteins. **B**. Quantification analysis of HDAC2, ♀ST+HDAC2.shRNA vs ♂ST+HDAC2.shRNA, ^####^ *p* < 0.0001. **C**. HSP90; ♀ST+HDAC2.shRNA vs ♂ST+HDAC2.shRNA, *p* = ns. **D**. SMN; ♀ST+HDAC2.shRNA vs ♂ST+HDAC2.shRNA; ****p < 0.0001. **E**. H3K27ac; ♀ST+HDAC2.shRNA vs ♂ST+HDAC2.shRNA; ***p < 0.001. **F**. H3K9ac; ♀ST+HDAC2.shRNA vs ♂ST+HDAC2.shRNA, **p < 0.01. **G**. OTR, ♀ST+HDAC2.shRNA vs ♂ST+HDAC2.shRNA, ***p < 0.001. **H**. OXT; ♀ST+HDAC2.shRNA vs ♂ST+HDAC2.shRNA, ****p < 0.0001. Molecular docking of protein–protein interaction. **I**. HDAC2-OTR, **J**. HDAC2-OXT. **K**. OXT-Acetyl-Histones H3 (Lys9), and **L**. OXT-Acetyl-Histones H3 (Lys27). **M**. OXT immunostaining. **N**. OXT^+^ cells; ♀ST+HDAC2.shRNA vs ♂ST+HDAC2.shRNA, ***p < 0.001. **O**. HDAC2/NeuN double immunostaining, **P**. HDAC2 positive cells in the cortex. ♀ST+HDAC2.shRNA vs ♂ST+HDAC2.shRNA, ^##^ *p* < 0.01. **Q**. HDAC2 positive cells in the striatum; ♀ST+HDAC2.shRNA vs ♂ST+HDAC2.shRNA, ^###^ *p* < 0.001. **R**. HSP90 immunostaining. **S**. HSP90 positive cells in the cortex, ♀ST+HDAC2.shRNA vs ♂ST+HDAC2.shRNA, ^##^ *p* < 0.01. **T**. HSP90 positive cells in the striatum, ♀ST+HDAC2.shRNA vs ♂ST+HDAC2.shRNA, ^#^ *p* < 0.05. *n* = 5. Data expressed as mean ± SEM.

To elucidate the mechanistic basis of HDAC2-OXT interactions, we performed in silico molecular docking studies (Figure 7I-L). Computational modeling revealed strong binding potential between HDAC2 and OXT (binding energy -8.2 kcal/mol), with particular affinity for the catalytic domain of HDAC2, suggesting direct regulatory interactions. This finding was corroborated by immunohistochemical analysis (Figure 7M-T and Figure S1A-B), which showed stroke-induced depletion of OXT-positive cells (65% reduction vs sham) alongside increased HDAC2-positive (3.1-fold) and HSP90-positive (2.7-fold) cells in both cortical and striatal regions. HDAC2 knockdown produced reciprocal effects, increasing OXT-positive cell density (particularly in females, 80% recovery vs sham) while reducing HSP90 expression (by 60% compared to stroke controls).

These coordinated findings from multiple experimental approaches consistently demonstrate that (1) ischemic stroke induces persistent epigenetic dysregulation through HDAC2 overexpression and histone deacetylation; (2) HDAC2 inhibition can effectively restore neuroprotective oxytocin signaling; and (3) female brains exhibit enhanced responsiveness to HDAC2 modulation, potentially due to estrogen-mediated facilitation of chromatin remodeling or sex-specific differences in stress response pathways. The strong molecular docking results further suggest that HDAC2 may directly regulate OXT expression through binding interactions, providing a mechanistic basis for their functional relationship in poststroke recovery. These findings position HDAC2 inhibition as a promising therapeutic strategy for stroke, with particular potential for addressing sex-specific recovery trajectories.

### 3.10 HDAC2 Knockdown in CPU Counteracts Oxidative Stress, Inflammation, and Neuronal Cell Death

We next evaluated the impact of HDAC2 knockdown on oxidative stress and mitochondrial damage in both sexes using dihydroethidium (DHE) staining for ROS detection and Tomm20/Cytochrome C (CytoC) double staining to assess mitochondrial integrity (Figure 8A-F). Quantitative analysis revealed a significant increase in ROS levels following stroke compared to sham controls in both cortical and striatal regions, with HDAC2 knockdown effectively reducing oxidative stress in both brain areas compared to stroke. Notably, female mice exhibited a more pronounced reduction in ROS levels following HDAC2 knockdown compared to males. Mitochondrial damage assessment showed a marked increase in CytoC^+^ Tomm20^+^ cells after stroke (indicating mitochondrial outer membrane permeabilization), which was significantly attenuated by HDAC2 knockdown, particularly in female animals. Western blot analysis of apoptosis-related proteins demonstrated elevated expression of pro-apoptotic markers (Bax, p53, and cleaved Caspase-3) in stroke groups (2.1–3.3 fold increase vs sham), with HDAC2 knockdown normalizing these levels more effectively in females (55–65% reduction vs stroke). Conversely, the antiapoptotic protein Bcl-2 showed decreased expression poststroke (40% reduction vs sham) that was restored by HDAC2 knockdown, with female mice demonstrating near-complete recovery (85% of sham levels, vs male). Analysis of inflammatory markers revealed significantly elevated levels of CRP, NSE, and cytokines (TNF-α, IL-6) in stroke animals (3.1–4.2 fold increase vs sham), which were reduced by HDAC2 knockdown to near-baseline levels in females (70–80% reduction vs stroke). Immunohistochemical validation confirmed these findings, showing increased NF-κB^+^ MAP2^+^ neurons (4.1 fold) and elevated CRP^+^/NSE^+^ NeuN^+^ cells in stroke groups that were markedly reduced by HDAC2 knockdown, particularly in female mice. These results demonstrate that HDAC2 knockdown effectively mitigates oxidative stress, preserves mitochondrial function, reduces apoptosis, and attenuates neuroinflammation following stroke, with consistently more robust effects observed in female animals, likely due to estrogen-enhanced epigenetic regulation and neuroprotective mechanisms.

**Figure 8:**
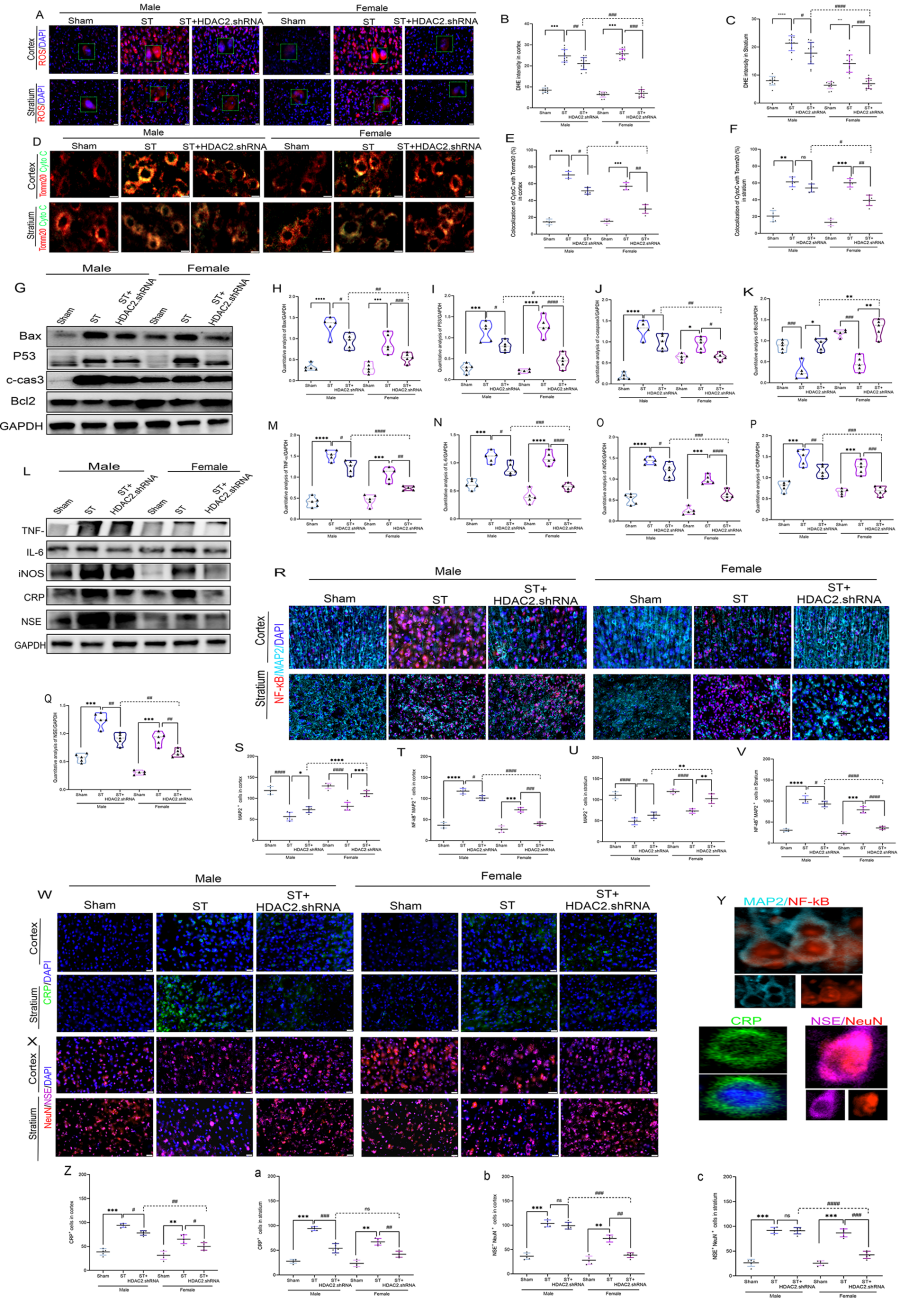
**HDAC2 knockdown in CPU counteracts oxidative stress, inflammation and, neuronal cell death. A**. ROS immunostaining. **B**. DHE intensity in the cortex; ♀ST+HDAC2.shRNA vs ♂ST+HDAC2.shRNA, ^###^ *p* < 0.001. **C**. DHE intensity in the striatum; ♀ST+HDAC2.shRNA vs ♂ST+HDAC2.shRNA, ^####^ *p* < 0.0001. **D**. CytoC Tomm20 double staining immunostaining. **E**. Colocalization of Cytoc with Tomm20 in the cortex; ♀ST+HDAC2.shRNA vs ♂ST+HDAC2.shRNA, ^#^ *p* < 0.05. **F**. Colocalization of Cytoc with Tomm20 in stratium;♀ST+HDAC2.shRNA vs ♂ST+HDAC2.shRNA, ^#^ *p* < 0.05. **G**. Western blot of apoptosis related proteins. Quantification analysis; of; **H**. Bax; ♀ST+HDAC2.shRNA vs ♂ST+HDAC2.shRNA, ^##^ *p* < 0.01. **I**. P53; ♀ST+HDAC2.shRNA vs ♂ST+HDAC2.shRNA, ^#^ *p* < 0.05. **J**. C-caspase3; ♀ST+HDAC2.shRNA vs ♂ST+HDAC2.shRNA, ^##^ *p* < 0.01. **K**. Bcl2; ♀ST+HDAC2.shRNA vs ♂ST+HDAC2.shRNA, **p < 0.01. **L**. Western blot of inflammatory related proteins. **M**. Quantification analysis; of TNF-α, ♀ST+HDAC2.shRNA vs ♂ST+HDAC2.shRNA, ^####^ *p* < 0.0001. **N**. IL-6; ♀ST+HDAC2.shRNA vs ♂ST+HDAC2.shRNA. ^###^ *p* < 0.001, **O**. iNOS:♀ST+HDAC2.shRNA vs ♂ST+HDAC2.shRNA,^###^ *p* < 0.001. **P**. CRP; ♀ST+HDAC2.shRNA vs ♂ST+HDAC2.shRNA, ^###^ *p* < 0.001. **Q**. NSE; ♀ST+HDAC2.shRNA vs ♂ST+HDAC2.shRNA. ^##^ *p* < 0.01. **R**. NF-kB MAP2 immunostaining. **S**. MAP2^+^ cells in the cortex, ♀ST+HDAC2.shRNA vs ♂ST+HDAC2.shRNA,****p < 0.0001,. **T**. NF-kB^+^MAP2^+^ cells in the cortex, ♀ST+HDAC2.shRNA vs ♂ST+HDAC2.shRNA, ^####^ *p* < 0.0001. **U**. MAP2^+^ cells in the striatum, ♀ST+HDAC2.shRNA vs ♂ST+HDAC2.shRNA, **p < 0.01. **V**. NF-kB^+^MAP2^+^ cells in the striatum,♀ST+HDAC2.shRNA vs ♂ST+HDAC2.shRNA, ^####^ *p* < 0.0001. **W**. CRP immunostaining. **X**. NSE^+^NeuN^+^ immunostaining. **Y**. Magnification of MAP2 NF-kB, CRP and NSE/NeuN scale bar 10 um. *n* = 5, scale bar 20 um. **Z**. CRP^+^ cells in cortex; ♀ST+HDAC2.shRNA vs ♂ST+HDAC2.shRNA, ^##^ *p* < 0.01, **a**. CRP^+^ cells in the striatum,♀ST+HDAC2.shRNA vs ♂ST+HDAC2.shRNA; *p* = ns. **b**. NSE^+^NeuN^+^ cells in the cortex; ♀ST+HDAC2.shRNA vs ♂ST+HDAC2.shRNA, ^###^ *p* < 0.001. **c**. NSE^+^NeuN^+^ cells in the striatum; ♀ST+HDAC2.shRNA vs ♂ST+HDAC2.shRNA, ^####^ *p* < 0.0001. Data expressed as mean ± SEM.

### 3.11 HDAC2 Knockdown in CPU Protects Organelles in Primary and Secondary Injured Sites

The cortical tissue ultrastructure undergoes profound pathological alterations following stroke, as demonstrated by cryogenic transmission electron microscopy (cryo-TEM) analysis in our experimental model (Figure 9A-N). These changes encompass multiple cellular and subcellular components, reflecting the complex pathophysiology of ischemic injury. Neuronal somata exhibit significant membrane disruption characterized by loss of plasma membrane integrity, pronounced indentations, and cytoplasmic swelling, while nuclei display chromatin margination and decomposition indicative of apoptotic processes. Mitochondria, critical for cellular energetics, undergo dramatic morphological changes including rounding of organelles, matrix swelling, and disintegration of cristae structure - alterations driven by calcium overload and reactive oxygen species accumulation. Axonal pathology manifests as detachment from the myelin sheath, neurofilament disorganization, and increased G-ratio, reflecting compromised structural integrity and impaired neurotransmission. The synaptic compartment shows substantial presynaptic terminal degeneration mediated by oxidative damage and microglial phagocytosis, while postsynaptic densities appear simplified. The Golgi apparatus, essential for protein processing and trafficking, demonstrates marked fragmentation of cisternae and loss of stacked organization, a consequence of ischemic stress disrupting vesicular transport. The neurovascular unit is similarly affected, with capillary endothelial cells showing swelling, tight junction disruption, and pericyte detachment, contributing to blood–brain barrier breakdown. Our ultrastructural analysis reveals that HDAC2 knockdown provides remarkable protection against these stroke-induced alterations. Treated animals show preserved neuronal membrane integrity, reduced chromatin condensation, and maintained cytoplasmic architecture. Mitochondrial morphology is significantly improved, with restoration of cristae density and elongation of organelles. Axonal pathology is attenuated, with normalized G-ratios and neurofilament organization. Synaptic preservation is evident through maintained presynaptic vesicle pools and postsynaptic density complexity. The Golgi apparatus demonstrates reorganization of cisternae and restoration of stacked morphology, while capillary endothelium shows reduced swelling and improved junction integrity. Notably, these neuroprotective effects exhibit sexual dimorphism, with female animals displaying more robust preservation of ultrastructure, potentially related to estrogen-mediated enhancement of HDAC2-dependent epigenetic regulation. The comprehensive protection afforded by HDAC2 inhibition spans multiple cellular compartments, suggesting its central role in coordinating the integrated response to ischemic injury through modulation of epigenetic landscapes, oxidative stress pathways, and cytoskeletal stabilization mechanisms. These findings position HDAC2 as a master regulator of ultrastructural integrity following stroke and highlight its therapeutic potential for preserving cellular architecture in the ischemic brain.

**Figure 9:**
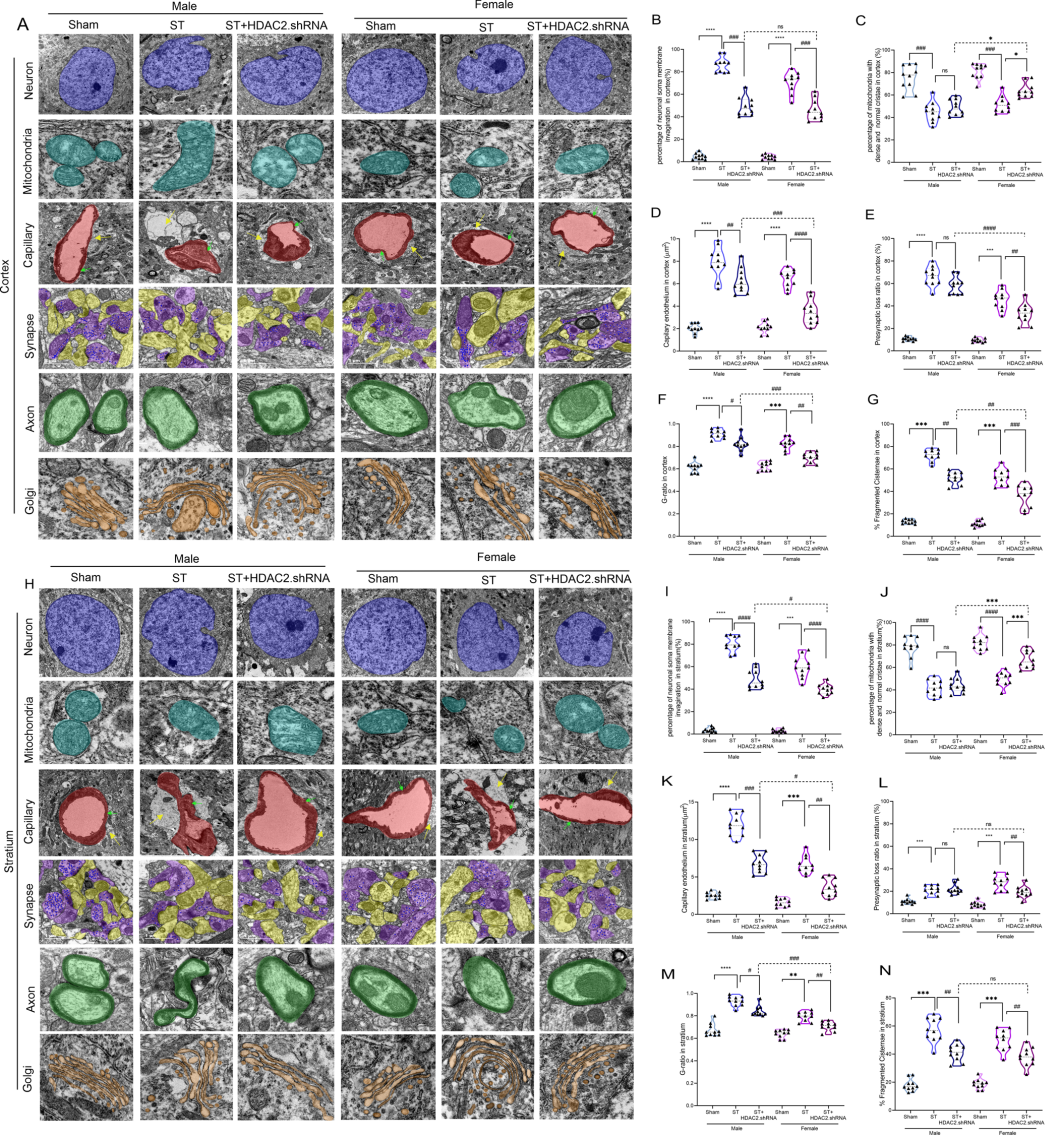
**HDAC2 knockdown in CPU protects organelles in primary and secondary injured sites**. **A**. Cortex organelles ultrastructure. Neuron, mitochondria, capillary, synapse, axon, golgi. **B**. Percentage of neuronal soma membrane invagination in cortex (%), ♀ST+HDAC2.shRNA vs ♂ST+HDAC2.shRNA: *p* = ns. **C**. percentage of mitochondria with dense and normal cristae in cortex (%), ♀ST+HDAC2.shRNA vs ♂ST+HDAC2.shRNA, *p < 0.05. **D**. Capillary endothelium in cortex, ♀ST+HDAC2.shRNA vs♂ST+HDAC2.shRNA: ^###^ *p* < 0.001. **E**. Presynaptic loss ratio in cortex (%); ♀ST+HDAC2.shRNA vs ♂ST+HDAC2.shRNA, ^####^ *p* < 0.0001. **F**. G-ratio in cortex, ♀ST+HDAC2.shRNA vs ♂ST+HDAC2.shRNA, ^###^ *p* < 0.001. **G**. % fragmented cisternae in cortex; ♀ST+HDAC2.shRNA vs ♂ST+HDAC2.shRNA, ^##^ *p* < 0.01. **H**. Striatum organelles ultrastructure. Neuron, mitochondria, capillary, synapse, axon, golgi. **I**. percentage of neuronal soma membrane invagination in the Striatum (%); ♀ST+HDAC2.shRNA vs ♂ST+HDAC2.shRNA, ^#^ *p* < 0.05. **J**. percentage of mitochondria with dense and normal cristae in Stratium (%), ♀ST+HDAC2.shRNA vs ♂ST+HDAC2.shRNA, ***p < 0.001. **K**. Capillary endothelium in the Striatum, ♀ST+HDAC2.shRNA vs ♂ST+HDAC2.shRNA: ^#^ *p* < 0.05. **L**. Presynaptic loss ratio in the Striatum (%); *p* = ns. **M**. G-ratio in the cortex, ♀ST+HDAC2.shRNA vs ♂ST+HDAC2.shRNA, ^###^ *p* < 0.001. **N**. % fragmented cisternae in the Striatum, ♀ST+HDAC2.shRNA vs ♂ST+HDAC2.shRNA: *p* = ns. *n* = 10. Data expressed as mean ± SEM.

### 3.12 HDAC2 knockdown increase synaptic plasticity, neuron survival and decrease inflammation via HDAC2/OXT signaling axis

Western blot analysis was performed to evaluate the impact of HDAC2 knockdown on the BDNF-TrkB-CREB signaling axis, a critical pathway for synaptic plasticity and neuronal survival, along with Synapsin I, a well-established presynaptic plasticity marker (Figure 10A–E). Our results demonstrated a significant downregulation of BDNF, TrkB, and pCREB expression in both male and female stroke models compared to sham controls, consistent with prior reports of stroke-induced synaptic dysfunction. Interestingly, HDAC2 knockdown restored expression levels of these proteins, with a more pronounced effect in females, suggesting potential sex-specific epigenetic regulation of neurotrophic signaling.

**Figure 10:**
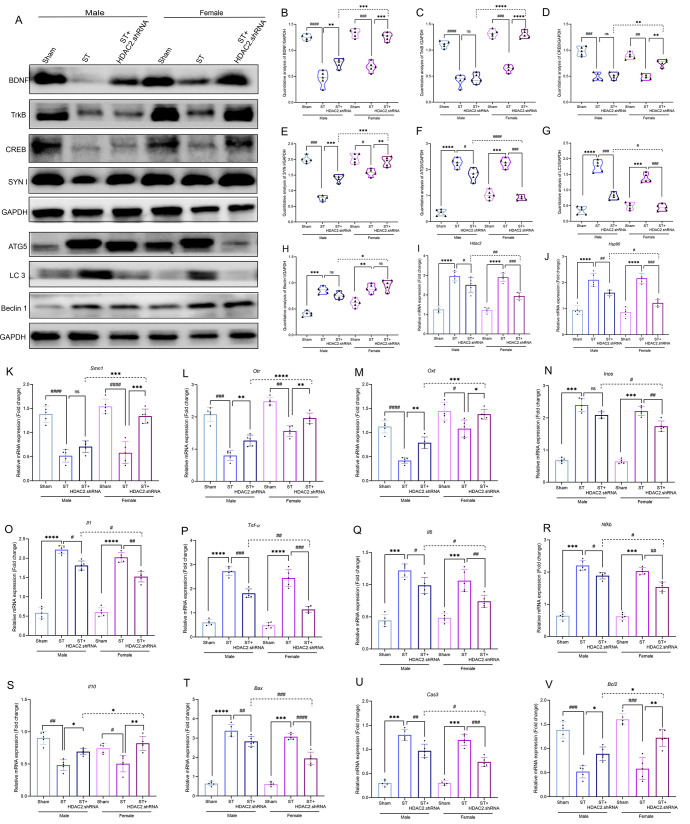
**HDAC2 knockdown increase synaptic plasticity, neuron survival and decrease inflammation via HDAC2/OXT signaling axis**. **A**. Western blot images. **B–H**. Quantification analysis of: **B**. BDNF; ♀ST+HDAC2.shRNA vs ♂ST+HDAC2.shRNA: ***p < 0.001. **C**. TrkB: ♀ST+HDAC2.shRNA vs ♂ST+HDAC2.shRNA:****p < 0.0001. **D**. CREB: ♀ST+HDAC2.shRNA vs ♂ST+HDAC2.shRNA, **p < 0.01. **E**. SYN I: ♀ST+HDAC2.shRNA vs ♂ST+HDAC2.shRNA: ***p < 0.001. **F**. ATG5; ♀ST+HDAC2.shRNA vs ♂ST+HDAC2.shRNA: ^####^ *p* < 0.0001. **G**. LC3; ♀ST+HDAC2.shRNA vs ♂ST+HDAC2.shRNA: ^#^ *p* < 0.05. **H**. Beclin1; ♀ST+HDAC2.shRNA vs ♂ST+HDAC2.shRNA: *p < 0.05. **I–V**. Relative mRNA expression: **I**. (*Hdac2)*, ♀ST+HDAC2.shRNA vs ♂ST+HDAC2.shRNA: ^##^ *p* < 0.01. **J**. (*Hsp90)*, ♀ST+HDAC2.shRNA vs ♂ST+HDAC2.shRNA: ^#^ *p* < 0.05. **K**. (*Smn1);* ♀ST+HDAC2.shRNA vs ♂ST+HDAC2.shRNA: ***p < 0.001. **L**. *(Otr)*, ♀ST+HDAC2.shRNA vs ♂ST+HDAC2.shRNA: ****p < 0.0001. **M**. *(Oxt);* ♀ST+HDAC2.shRNA vs ♂ST+HDAC2.shRNA:***p < 0.001. **N**. *(Inos);* ♀ST+HDAC2.shRNA vs ♂ST+HDAC2.shRNA: ^#^ *p* < 0.05. **O**. *(Il1);* ♀ST+HDAC2.shRNA vs ♂ST+HDAC2.shRNA: ^#^ *p* < 0.05, **P**. *(Tnf-α);* ♀ST+HDAC2.shRNA vs ♂ST+HDAC2.shRNA: ^##^ *p* < 0.01, **Q**. (*Il6);* ♀ST+HDAC2.shRNA vs ♂ST+HDAC2.shRNA: ^#^ *p* < 0.05*:* **R**. *(Nfkb);* ♀ST+HDAC2.shRNA vs ♂ST+HDAC2.shRNA, ^#^ *p* < 0.05. **S**. (*Il10);* ♀ST+HDAC2.shRNA vs♂ST+HDAC2.shRNA: *p < 0.05. **T**. (*Bax);* ♀ST+HDAC2.shRNA vs ♂ST+HDAC2.shRNA: ^###^ *p* < 0.001. **U**. *(Cas3);* ♀ST+HDAC2.shRNA vs ♂ST+HDAC2.shRNA: ^#^ *p* < 0.05. **V**. *(Bcl2);* ♀ST+HDAC2.shRNA vs ♂ST+HDAC2.shRNA: *p < 0.05. *n* = 5. Data expressed as mean ± SEM.

To investigate whether HDAC2 modulates poststroke autophagy, we analyzed key autophagy-related proteins (ATG5, LC3-II, and Beclin1) (Figure 10A, F–H) and LC3 positive cells by using immunostaining (Figure S6C-E). Stroke triggered a pathological upregulation of ATG5 and LC3-II (indicative of increased autophagosome formation), whereas HDAC2 knockdown significantly attenuated these changes in both sexes but more marked in females. Notably, Beclin1 levels remained unchanged across all groups, implying that HDAC2 inhibition selectively disrupts autophagosome maturation downstream of Beclin1-mediated initiation. This aligns with evidence that HDAC2 regulates ATG5 expression via histone deacetylation at the ATG5 promoter.

qPCR validation further corroborated these findings (Figure 10 I–V). Stroke upregulated pro-inflammatory *(Tnf-α, Il6, Il1β, Nfkb, Inos*) and pro-apoptotic genes (Casp3, Bax), while downregulating neuroprotective genes (Oxt, Otr, Smn1, Il10, Bcl2). HDAC2 knockdown reversed these expression patterns, with females exhibiting more robust recovery a phenomenon potentially linked to estrogen-enhanced chromatin remodeling. The concordance between qPCR and Western blot data underscores the reproducibility of our observations.

## 4 Discussion

Ischemic stroke remains a leading cause of long-term disability and mortality worldwide, with current therapeutic strategies offering only limited neuroprotection.^33^ The majority of preclinical stroke studies have utilized male mice, with female studies being underrepresented, despite significant sex differences in stroke outcomes. These differences arise from variations in infarct location, hormonal influences, and differential cellular responses to ischemia. Recent advances in epigenetic research have highlighted histone deacetylases (HDACs), particularly HDAC2, as critical regulators of gene expression in neurological disorders.^34^

Our study demonstrates that HDAC2 knockdown confers significant neuroprotection in ischemic brain injury, with sex-specific effects mediated through the oxytocin (OXT) signaling pathway.

Our in vitro data demonstrate that HDAC2 inhibition protects neurons from ischemic death. This effect was confirmed in both female-derived human neuroblastoma (SH-SY5Y) cells and male-derived mouse hippocampal (HT-22) neurons, indicating that the core cytoprotective mechanism is conserved across sex and species in these models.^35^ These findings align with previous studies showing that HDAC2 inhibitors mitigate ischemic damage by promoting antiapoptotic and anti-inflammatory pathways, including the upregulation of Bcl-2 and suppression of pro-inflammatory cytokines such as TNF-α.^36,37^ *In vivo* experiments using endothelin-1 (ET-1)-induced ischemia showed that HDAC2 knockdown reduces infarct volume, improves cerebral blood flow, and enhances behavioral outcomes, consistent with reports that epigenetic modulation influences stroke recovery.^38^ Notably, female mice exhibited more pronounced neuroprotection, potentially due to interactions between HDAC2 and estrogen. Our data show equivalent initial injury using the ET-1 model; however, potential sex differences in endothelin receptor signaling or vasoconstrictive profiles warrant further investigation.

HDAC2 is a histone deacetylase involved in epigenetic regulation, exacerbates neuronal susceptibility to ischemic injury. Studies have shown that HDAC2 upregulation increases neuronal damage postischemia, while HDAC2 deficiency or knockdown confers neuroprotection.^39,40^ For instance, HDAC2 knockdown in cultured neurons restores protective proteins, such as Endophilin-B1 (Endo-B1b/c), reducing mitochondrial damage, caspase activation, and neuronal death following ischemic insult or beta-amyloid toxicity.^40,41^ *In vivo*, HDAC2 deficiency significantly reduces infarct size and improves neuronal survival in stroke models.^42,43^ Moreover, HDAC2 suppression during a critical poststroke window (5–7 days post- ischemia) promotes functional recovery by enhancing neuroplasticity and reducing neuroinflammation, effects not observed with inhibition of other HDAC isoforms, such as HDAC1 or HDAC3.^42^ These findings establish HDAC2 as a pivotal mediator of ischemic injury and a promising therapeutic target.

Our mechanistic experiments using ovariectomy and pharmacological agents strongly support the central hypothesis that estrogen is a primary mediator of neuroprotection in females. The exacerbation of stroke injury following OVX or aromatase inhibition, and its rescue by exogenous estradiol, provides direct causal evidence for the protective role of endogenous estrogen in females, aligning with clinical observations of increased stroke risk postmenopause,^44^ The finding that oxytocin receptor antagonism worsened outcomes in both sexes, but oxytocin release suppression specifically harmed females, suggests a complex interaction between estrogen and the oxytocin system. We propose a model where basal estrogen levels in females potentiate oxytocin signaling, which in turn activates downstream survival pathways.^45^ The [neutral/moderate] effect of progesterone suggests its role may be secondary or dependent on the presence of estrogen.^46,47^ Importantly, the ability of SHR1653 to worsen injury in males indicates that oxytocin receptor signaling also confers baseline protection in the male brain, albeit potentially through different regulatory mechanisms. These results highlight that the sexually dimorphic response to stroke is not merely due to the presence or absence of estrogen but involves a layered interaction between sex hormones and neuropeptide systems, offering multiple potential targets for future sex-specific therapeutic strategies.

Oxytocin (OXT) and its receptor (OTR) are critical modulators of neuroprotection in ischemic stroke. OXT inhibits glutamate release and blocks the NR2B/CREB signaling pathway, promoting neuronal survival and mitigating ischemic injury-induced cognitive decline.^48^ The finding that HDAC2 knockdown increases OXT and OTR expression, particularly in female brains, suggests that HDAC2 negatively regulates the OXT pathway. Activation of OXT signaling reduces inflammation and oxidative stress while enhancing neuronal survival, indicating that OXT is a key downstream effector of HDAC2 modulation. This aligns with reports that oxytocin’s neuroprotective effects involve anti-inflammatory actions and reduction of excitotoxicity, with limited efficacy in severe ischemia or in aged animals, highlighting the need for further investigation into its therapeutic window.^49,50^

The clinical implications of this study are significant, suggesting that HDAC2 inhibition and OXT pathway activation could serve as novel therapeutic strategies for ischemic stroke. HDAC inhibitors, such as valproic acid and sodium butyrate, have shown neuroprotective potential in preclinical models,^51^ and their repurposing for stroke treatment warrants further investigation. Additionally, OXT analogs (e.g., carbetocin) or OTR agonists could be investigated as adjunct therapies, particularly in female patients where the response appears stronger. However, a critical limitation is the underrepresentation of female subjects in stroke studies, necessitating sex-stratified clinical trials.^47^ Future research should explore whether blood OXT levels could serve as a prognostic biomarker for stroke recovery and whether combination therapies targeting both HDAC2 and OXT pathways yield synergistic benefits.

The interplay between estrogen, HDAC2, and oxytocin signaling is central to the observed Sex-specific neuroprotection. Estrogen not only downregulates HDAC2 but also modulates astrocyte function and inflammatory responses in the ischemic brain, contributing to female resilience against ischemic injury.^48^ Astrocytes in females exhibit higher estrogen receptor expression and greater resistance to oxidative stress, partly due to estrogen’s regulation of gene expression through epigenetic mechanisms involving HDACs. Although initial GFAP upregulation was similar between sexes, the functional consequences of astrocyte reactivity are known to differ. Female astrocytes typically exhibit a more protective phenotype, supported by estrogen. Critically, our observed changes in AQP4 a key regulator of poststroke edema and glymphatic clearance alongside GFAP at Day 21 suggest that HDAC2 inhibition may be differentially influencing these sex-divergent repair processes. At this chronic phase of glial scar maturation, treatment-induced shifts in the GFAP/AQP4 axis could modify scar properties, potentially reducing its inhibitory nature in males or enhancing its supportive functions in females, thereby contributing to the sex-specific recovery patterns observed.^52–54^

Additionally, estrogen’s modulation of histone acetylation facilitates gene expression changes that enhance OXT signaling. The suggestion that HDAC2 knockdown activates the OXT pathway “potentially through interactions with estrogen” is supported by evidence that estrogen regulates both HDAC2 levels and epigenetic states conducive to neuroprotection.^55,56^ This triangular relationship could explain the stronger neuroprotective effects of HDAC2 inhibition in females. The study underscores the importance of sex-specific considerations in stroke treatment strategies. Current therapies provide limited neuroprotection, and the identification of HDAC2 as a modifiable factor with sex-specific effects opens new therapeutic avenues. Targeting HDAC2 to enhance OXT signaling, particularly in females, may improve outcomes by reducing infarct size, inflammation, and oxidative stress while promoting neuronal survival and functional recovery. Moreover, understanding estrogen’s role in modulating HDAC2 and OXT pathways may enable tailored approaches based on hormonal status, age, and sex, advancing personalized stroke management.^48,49^

### 4.1 Translational Considerations

A key translational consideration arising from our study is the estrogen-dependent nature of the observed neuroprotection. Our ovariectomy and aromatase inhibition experiments demonstrate that the full therapeutic benefit of HDAC2 inhibition in females requires endogenous estrogen. This finding has direct implications for clinical populations, as a substantial proportion of female stroke patients are postmenopausal and thus estrogen-deficient. While this might suggest a limited therapeutic window in older women, several avenues warrant future investigation. First, it is possible that estrogen-priming or short-term estradiol replacement could resensitize the oxytocinergic pathway to HDAC2 inhibition in a safe, acute poststroke context, though this would require careful evaluation of cardiovascular risks. Second, the protective pathway might be activated by targeting downstream effectors, such as direct oxytocin receptor agonists (e.g., carbetocin), which could bypass the need for estrogen to amplify OXT signaling. Finally, the modest but significant benefits observed in male mice in our study suggest that HDAC2 inhibitors may still offer some neuroprotection in low-estrogen states, albeit through potentially different downstream mechanisms. These considerations underscore the need for age- and hormone-stratified preclinical studies and clinical trial designs to effectively translate this sex-specific pathway into practice.^57,58^

## 5 Limitations

While the ET-1 model provides a reliable and reproducible method for inducing focal ischemia with low mortality, we acknowledge its inherent limitations. ET-1 acts directly on endothelin receptors expressed on neurons, glia, and vascular cells, meaning its effects are not restricted solely to vasoconstriction and ischemia induction.^59^ These direct receptor-mediated effects could potentially influence downstream cellular responses independently of the ischemic cascade. Furthermore, while we observed equivalent infarct volumes and CBF reduction in male and female mice at baseline using this model, this finding contrasts with some other stroke models (e.g., MCAO) where sex differences in acute injury severity are often reported.^60–62^ The absence of baseline sex differences in our study allows for a clean interpretation of the sex-dependent therapeutic effects of HDAC2 inhibition, as later outcomes are not confounded by unequal initial injuries. However, this model-specific characteristic should be considered when generalizing these findings, and future studies using complementary models are warranted to confirm the broader applicability of the HDAC2-OXT mechanism.

## 6 Conclusions

This study provides compelling evidence that sex-specific HDAC2 knockdown alleviates ischemic brain injury by activating the OXT/OTR pathway, with more robust effects in females. These findings enhance our understanding of stroke pathophysiology and have the way for personalized, sex-specific stroke therapies. Future studies should focus on translating these findings into human trials, elucidating the molecular crosstalk between HDAC2, OXT, and estrogen signaling, and addressing potential limitations of HDAC inhibitors, such as off-target effects. By integrating epigenetic and neuropeptide-based approaches, this research opens new avenues for improving outcomes in ischemic stroke patients.

## Supplementary Material



## Data Availability

The authors declare that all data supporting the findings of this study are available within the article and its Supporting Information or from the corresponding author upon reasonable request.

## Notes

Additional experimental details, photographs of model procedures, behavioral tests, protein expression analysis, immunofluoroscence data, gene expression analysis, antibodies information, observed genes, and list of predicted proteins (PDF)

## Abbreviations

(HDAC2): Histone deacetylase 2
(OXT): oxytocin
(OTR): oxytocin receptor
(OGD/R): oxygen-glucose deprivation/reoxygenation
(ET-1): endothelin-1
(L-NAME): Nω-Nitro-l-arginine methyl ester
(AAV9): Viral vectors including adeno-associated virus serotype 9
(shRNA): delivering short hairpin RNA
(LSCI): laser speckle contrast imaging
(ROS): reactive oxygen species
(DHE): dihydroethidium
(TEM): transmission electron microscopy
(AQP4): aquaporin-4
(eNOS): endothelial nitric oxide synthase
(nNOS): neuronal nitric oxide synthase
(GFAP): glial fibrillary acidic protein
(Iba-1): ionized calcium-binding adapter molecule 1
(BDNF): brain-derived neurotrophic factor
(TrkB): tropomyosin receptor kinase B
(CREB): cAMP response element-binding protein
(NF-κB): nuclear factor kappa-light-chain-enhancer of activated B cells
(TNF-α): tumor necrosis factor-alpha
(IL-6): interleukin-6
(IL-1β): and interleukin-1 beta
(LC3): microtubule-associated protein 1*A*/1B-light chain 3
(ATG5): autophagy-related protein 5
(Bcl-2): B-cell lymphoma 2
(Bax): Bcl-2-associated X protein
(Casp3): caspase-3
(H3K27ac): histone H3 lysine 27 acetylation
(H3K9ac): lysine 9 acetylation
(HSP90): heat shock protein 90
(SMN): survival motor neuron protein
(CPU): caudate-putamen
(ER): estrogen receptor
(PI3K): phosphatidylinositol 3-kinase
(Akt): Protein kinase B
(Tomm20): Translocase Of Outer Mitochondrial Membrane 20
(cyto) c: Cytochrome C
(SYN I): Synapsin-1.
