# Supplementary material for: An Epigenetic Switch for Sex-Specific Brain Resilience in Stroke: Targeting HDAC2 to Amplify Endogenous Oxytocin Signaling
Source: ACS Cent Sci. 2026 Mar 25;12(4):497–523. doi: 10.1021/acscentsci.6c00191 (PMC13107219; doi:10.1021/acscentsci.6c00191)
Supplement: Supplementary file 1 [file oc6c00191_si_001.pdf]

Supporting Information for Publication

An Epigenetic Switch for Sex-Specific Brain Resilience in Stroke: Targeting HDAC2 to Amplify Endogenous Oxytocin Signaling

Nashwa Amin [1,2,3,4], Xia Yuan [1,2], Zongjie Shi[4], Fei Wu [1,2], Irum Naz Abbasi [1,2], Yang Yang[5], Suhong Ye[6], Qining Yang [7], Yu Geng[4**], Marong Fang[1,2,*]**

[1]Department of Orthopedics of Children's Hospital, Zhejiang university school of medicine, National Clinical Research Center For Children and Adolescents' Health and Diseases, 310052, Hangzhou, China.

[2]Institute of System Medicine, Zhejiang University School of Medicine, Zhejiang University, 310058, Hangzhou, China

[3]Department of Zoology, Faculty of Science, Aswan University, 81521, Aswan, Egypt.

[4]Center for Rehabilitation Medicine, Department of Neurology, Zhejiang Provincial People's Hospital, Affiliated People's Hospital, Hangzhou Medical College, 310014, Hangzhou, China.

[5]Faculty of Medicine, Macau University of Science and Technology, Macau, 999078, Taipa, China.

[6]Department of Neurology and Psychiatry, The Second Hospital of Jinhua, 321004, Jinhua,China.

[7]Department of Orthopaedics, Jinhua Municipal Central Hospital, Affiliated Jinhua Hospital,

Zhejiang University School of Medicine, 321000, Jinhua, China.

* Correspondence:

Marong Fang, PhD, Department of Orthopedics of Children's Hospital, Zhejiang university school of medicine, National Clinical Research Center For Children and Adolescents' Health and Diseases, Hangzhou 310052, China. E-mail addresses: fangmaro@zju.edu.cn [M. Fang]. ORCID: 0000-0002-6636-4347

**Co-Correspondence:

Qining Yang, MD, Jinhua Municipal Central Hospital, Affiliated Jinhua Hospital, Zhejiang University School of Medicine, Jinhua 321000, China, E-mail addresses: jhyangqn@163.com. ORCID: 0009-0001-0804-7618.

Yu Geng, MD, Center for Rehabilitation Medicine, Department of Neurology, Zhejiang Provincial People's Hospital, Affiliated People's Hospital, Hangzhou Medical College, Hangzhou, Zhejiang 310014, China. E-mail addresses: gengyu@hmc.edu.cn.

Supplementary Figures

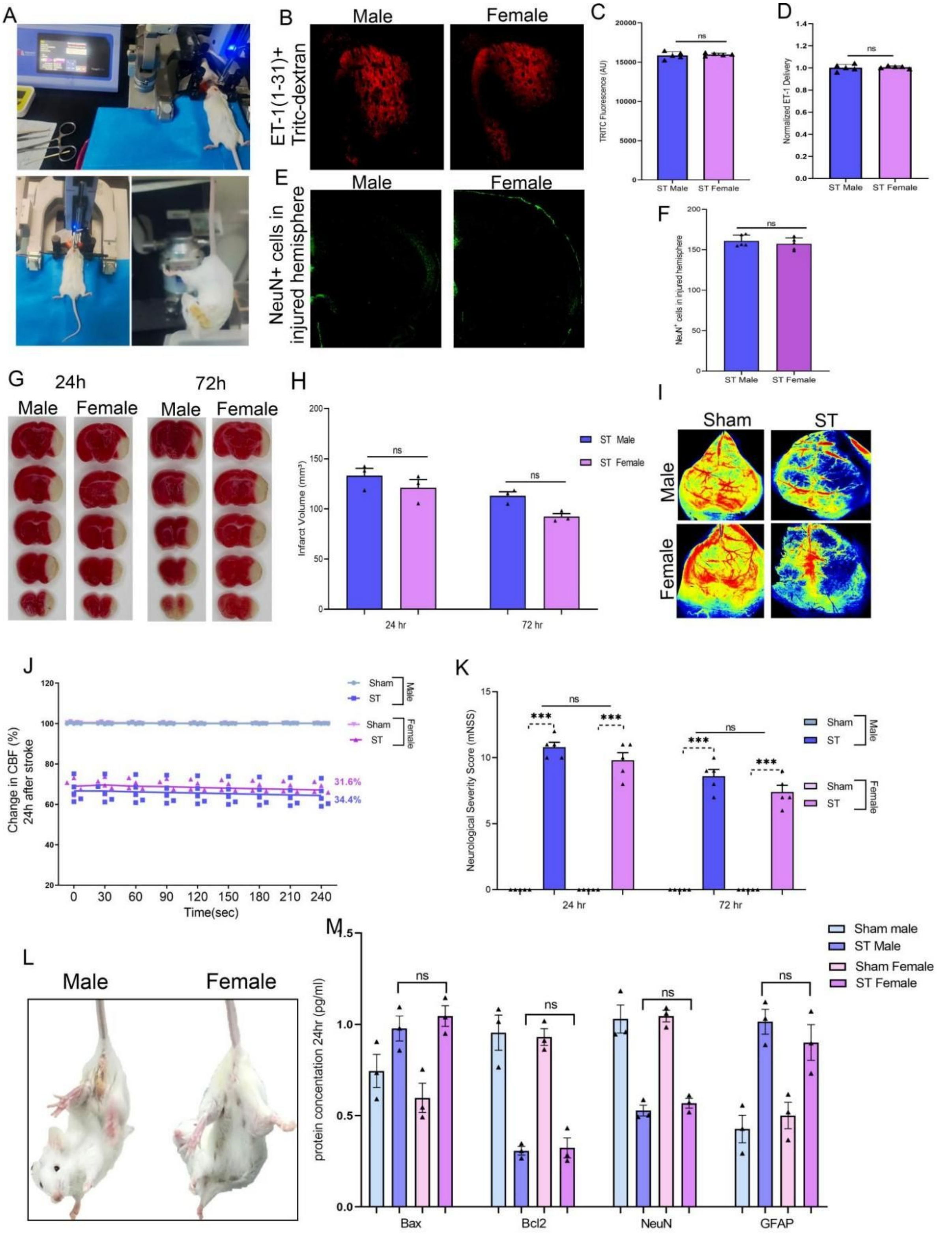

Figure S1. Validation of Equivalent Stroke Induction and Baseline Severity between Sexes. **A**. Model procedures. Correlation between TRITC fluorescence (ET-1 delivery) and initial infarct volume at 24 hours, to control for variability in vasoconstrictor delivery, ET-1(1-31) was co-

injected with the fluorescent tracer TRITC-dextran into the motor cortex: **B.** ET-1+TRITC-dextran **C**. TRITC fluorescence, **D**. Normalization of ET-1 delivery ♂ST vs. ♀ST; *p= ns, n=5*,**E.** NeuN immunostaining**, F.** NeuN positive cells ♂ST vs. ♀ST; *p= ns, n=5*, **G.** Representative TTC-stained brain sections showing infarct areas (white) at 24&72 hours post-stroke., **H**. Infarct volumes measured by TTC staining at 24 and 72 hours post-stroke ♂ST vs. ♀ST; *p= ns,n=3*, **I**.Representative cerebral blood flow (CBF) maps at 24 hours post-stroke in male and female mice, **J**. Change in CBF % ♂ST vs. ♀ST; *p= ns, n=5*, **K**.Neurological deficit mNSS assessed at 24 and 72 hours post-stroke♂ST vs. ♀ST; *p= ns, n=5*, **L**. Male & female neurological defict, **M.** Baseline Bax, Bcl2, NeuN and GFAP proteins concentration 24hr after model in male and female♂ST vs. ♀ST; *p= ns, n=3*. This comprehensive set of control experiments demonstrates that male and female mice experienced strokes of equivalent initial severity. Identical ET-1 delivery , comparable neuronal survival, similar infarct volumes, equivalent CBF reduction, and comparable early neurological deficits establish that any sex differences observed in later recovery parameters are attributable to divergent post-stroke recovery mechanisms rather than differences in initial injury severity. All comparisons between male and female stroke groups showed no significant difference. Data are presented as mean ± SEM.

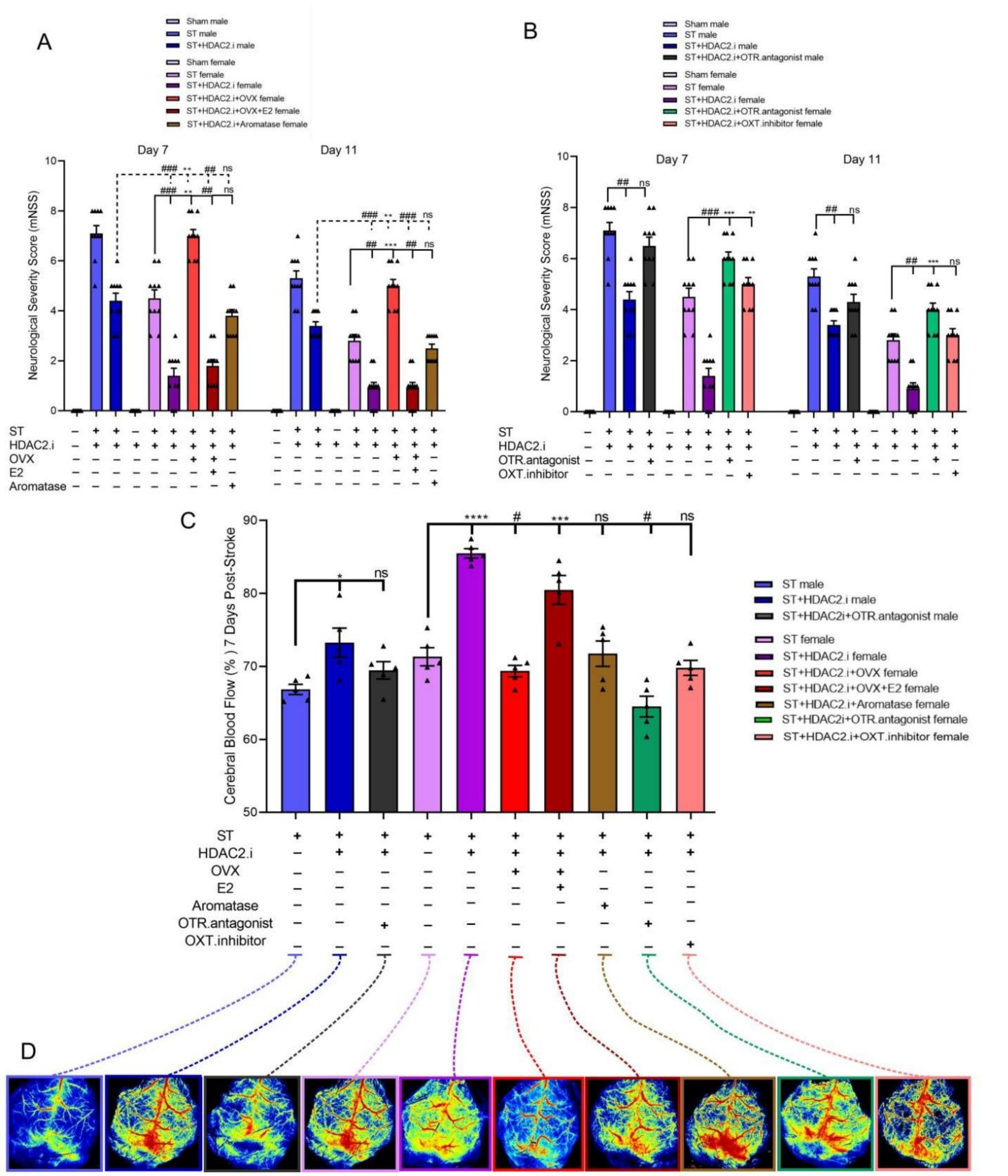

Figure.S2.Effects of HDAC2 inhibition, estrogen manipulation, and oxytocin pathway blockade on neurological function and cerebral blood flow after stroke. A, B. Neurological Severity Scores (mNSS) at Day 7 and Day 11 post-stroke in different experimental groups, Higher scores indicate greater neurological impairment., n=10. Data are presented as mean ± SEM. **A.** Groups include Stroke (ST), ST + HDAC2 inhibitor (HDAC2.i), ST + OVX, ST + OVX + E2, and

ST + Aromatase. **B**. Groups include ST, ST + HDAC2.i, ST + OTR antagonist (OTR.antagonist), and ST + OXT inhibitor (OXT.inhibitor). **C**. Cerebral blood flow (% change from baseline) measured over 7 days post-stroke across treatment groups, n=5. Data are presented as mean ± SEM.: ST, ST + HDAC2.i, ST + OVX, ST + OVX + E2, ST + Aromatase, ST + OTR.antagonist, and ST + OXT.inhibitor. Data are presented as mean ± SEM. **D.** Laser speckle imaging. Abbreviations: mNSS, modified Neurological Severity Score; OVX, ovariectomy; E2, 17β-estradiol; OTR, oxytocin receptor; OXT, oxytocin. HDAC2 inhibition significantly improves neurological function and CBF in both sexes, but the effect is significantly greater in females (♀ST+HDAC2.i vs. ♂ST+HDAC2.i). Ovariectomy (OVX) abolishe the protective effects of HDAC2.i in females (♀ST+HDAC2.i vs. ♀ST+HDAC2.i+OVX). Estradiol is sufficient: Estradiol replacement (E2) completely rescues the protective phenotype in OVX females (♀ST+HDAC2.i+OVX vs. ♀ST+HDAC2.i+OVX+E2).Oxytocin signaling is required in females: Blockade of the oxytocin receptor (OTR.antagonist) or oxytocin release (OXT.inhibitor) reverses HDAC2.i-mediated protection in females (♀ST+HDAC2.i vs. ♀ST+HDAC2.i+OTR.antagonist).Sex-specific OTR reliance: OTR antagonism has minimal effect in males (♂ST+HDAC2.i vs. ♂ST+HDAC2.i+OTR.antagonist), revealing a fundamental sex difference in pathway dependence. These data provide direct causal evidence that the superior neuroprotective efficacy of HDAC2 inhibition in females is strictly dependent on estrogen, which in turn enables a protective program requiring intact oxytocin signaling.

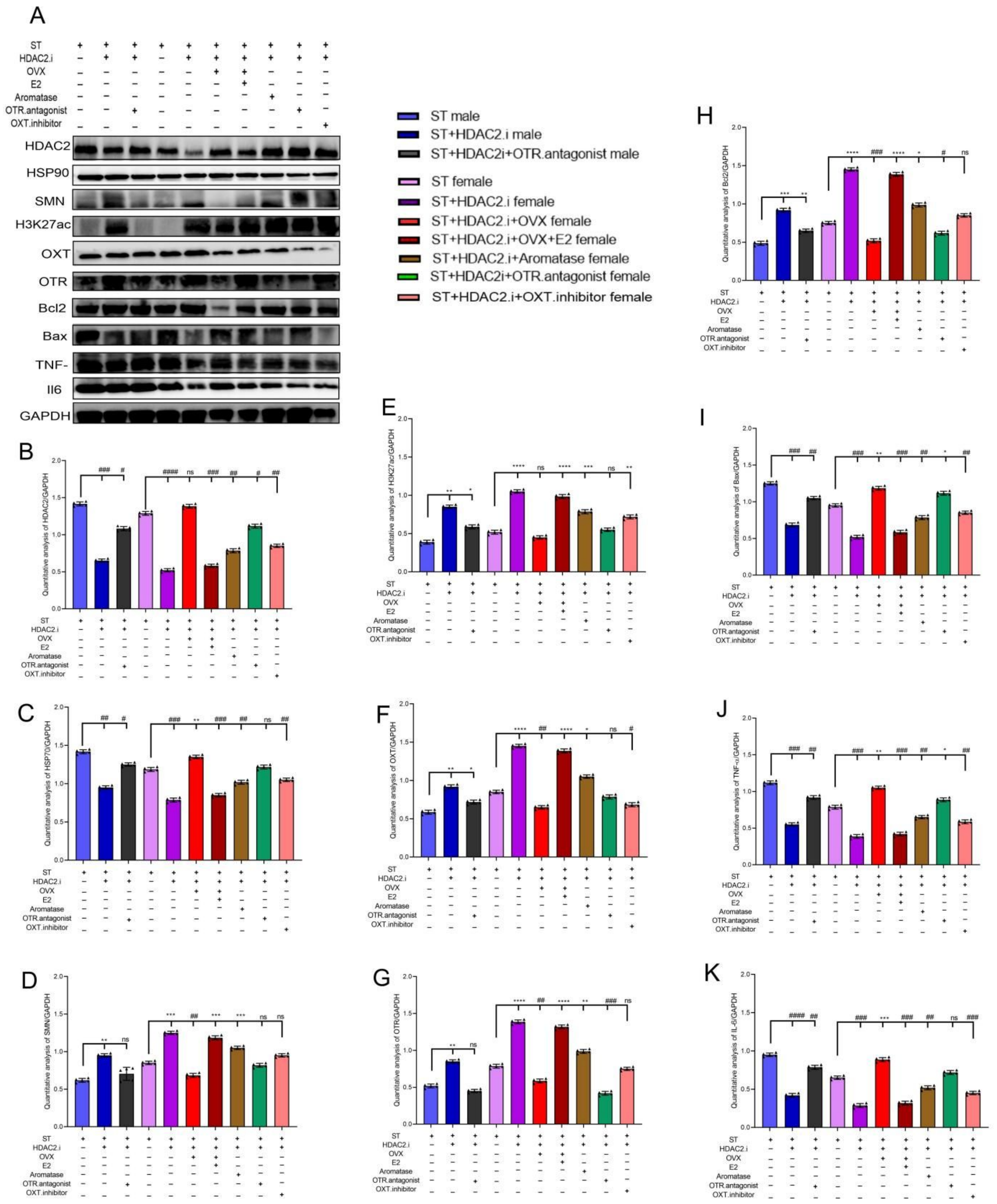

Figure S3. Quantitative protein expression data across experimental groups confirm the estrogen-dependent regulation of the HDAC2-OXT neuroprotective axis. A. Western blot represent individual proteins**.** Quantification analysis; **B.** HDAC2**, C.** HSP90**, D.**SMN**, E.** H3K27ac**, F.**OXT**, G.**OTR, **H.** Bcl2**, I.**Bax, **J**. TNF-α, **K**.IL-6. Sham Treatment (ST), HDAC2 inhibitor

(HDAC2i), Ovariectomy (OVX), Estradiol replacement (E2), Aromatase inhibition (Aromatase), Oxytocin Receptor antagonist (OTR antagonist), and Oxytocin inhibitor (OXT inhibitor). Values are normalized fold-changes relative to the ST group. The data illustrate distinct proteomic profiles induced by estrogen manipulation (OVX, E2, Aromatase inhibition) and oxytocin pathway blockade, supporting the modulation of key signaling pathways discussed. The protective molecular signature induced by HDAC2.i in females was largely abolished by estrogen depletion. In the ♀ST+HDAC2.i+OVX vs. ♀ST+HDAC2.I group, Bcl2 levels decreased $^{###}$*p<0.001* , while Bax , TNF-α, and IL-6 increased ***p<0.01*,***p<0.01*, ****p<0.001* reverting to a pathological profile. Crucially, estradiol replacement in ovariectomized females (♀ST+HDAC2.i+OVX+E2) completely restored the protective molecular profile. Bcl2 levels were restored to those seen in the intact ♀ST+HDAC2.i group (♀ST+HDAC2.i+OVX vs. ♀ST+HDAC2.i+OVX+E2: *****p<0.0001* for Bcl2), providing direct causal evidence that estradiol is the key ovarian factor. Blockade of the oxytocin receptor in females receiving HDAC2.i (♀ST+HDAC2.i+OTR-antagonist) also reversed the protective molecular effects. This group showed significantly elevated Bax (♀ST+HDAC2.i vs. ♀ST+HDAC2.i+OTR-antagonist: **p<0.05*) and inflammatory cytokines, phenocopying the effect of estrogen depletion, n=5. Data are presented as mean ± SEM.

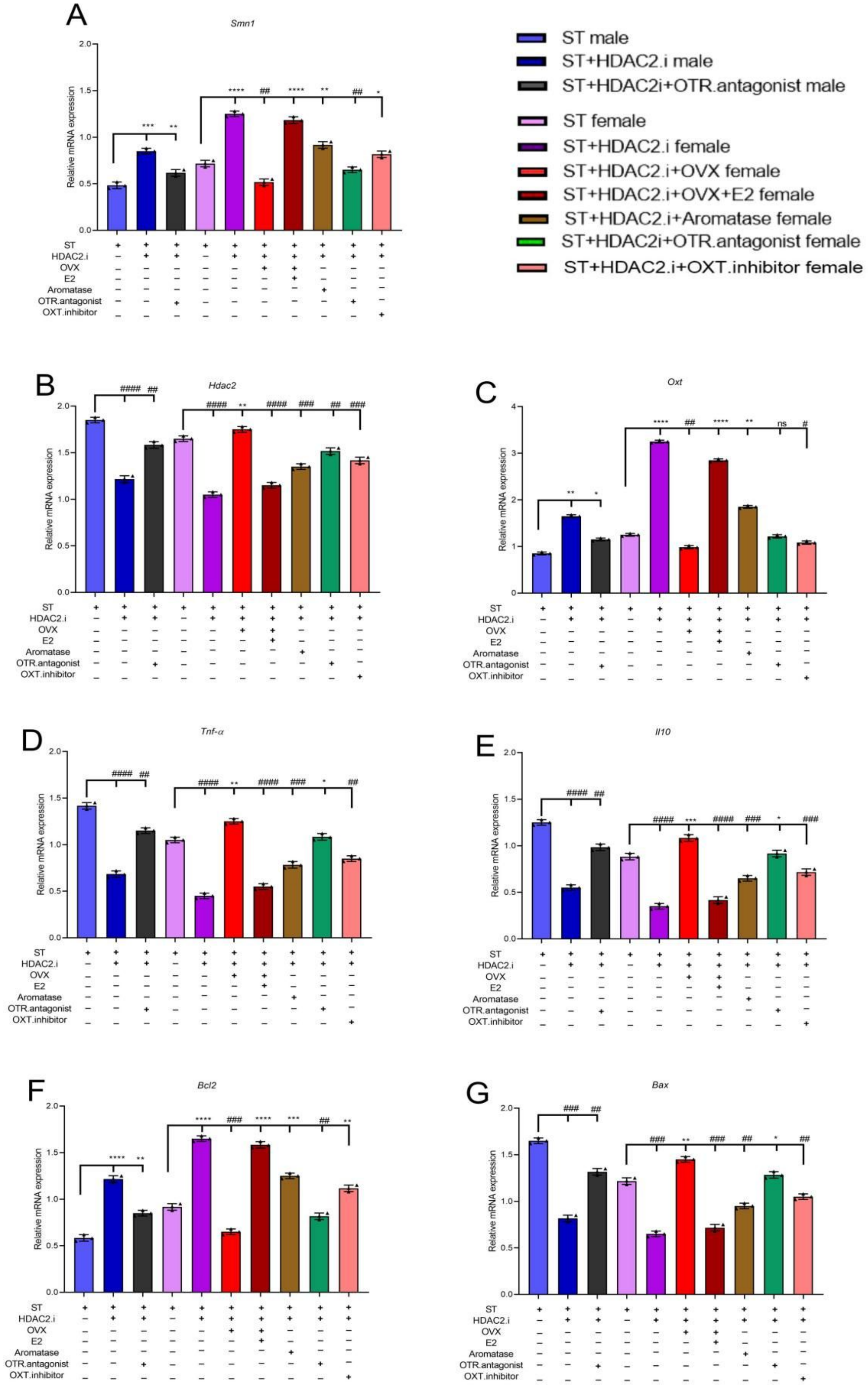

Figure. S4.Gene expression analysis confirms the estrogen-dependent regulation of the HDAC2-OXT neuroprotective axis. A-G. Gene expression analysis. Relative mRNA expression

levels normalized to β-actin and are presented as fold-change relative to the male Stroke (ST) group for key genes involved in apoptosis, inflammation, and stress response, n=3. Data are presented as mean ± SEM; **A**. *Smn1,* **B.** *Hdac2*, **C.** *Oxt,* **D.** *TNF-α,* **E**. IL-10, **F**. *Bcl2*, **G**. *Bax*, . Data are presented as mean ± SEM. The patterns demonstrate the regulatory effects of estrogen status (OVX, E2, Aromatase inhibition) and oxytocin pathway blockade (OTR antagonist, OXT inhibitor) on post-stroke gene expression. Ovariectomy (OVX) reversed the protective gene expression profile in females, decreasing Oxt; $^{\#\#}p<0.01$,and Bcl2 ;$^{\#\#}p<0.01$ while increasing Tnf-α; $^{**}p<0.01$and Il-10;$^{***}p<0.001$ (♀ST+HDAC2.i vs. ♀ST+HDAC2.i+OVX). Estradiol replacement (E2) completely restored the protective profile (♀ST+HDAC2.i+OVX vs. ♀ST+HDAC2.i+OVX+E2). OTR antagonism or OXT inhibition in females receiving HDAC2.i phenocopied estrogen loss, partially block the effect of HDAC2 inhibitor in controlling in these genes; Tnf-α, Il-6, and Bax,Oxt and Bcl2 (♀ST+HDAC2.i vs. ♀ST+HDAC2.i+OTR.antagonist; ♀ST+HDAC2.i vs. ♀ST+HDAC2.i+OXT.inhibitor). In males, OTR antagonism (♂ST+HDAC2.i+OTR.antagonist) had minimal effect on gene expression compared to HDAC2.i alone, contrasting sharply with the dramatic effects observed in females. This gene expression data provides comprehensive mechanistic validation that the superior neuroprotective effect of HDAC2 inhibition in females is strictly estrogen-dependent and requires intact oxytocin signaling. The loss of protection following OVX, aromatase inhibition, or OTR blockade and its rescue by estradiol establishes a defined hormonal epigenetic mechanism (Estrogen induce Oxytocin/OTR Signaling led to HDAC2 modulation) underlying neuroprotection after stroke.

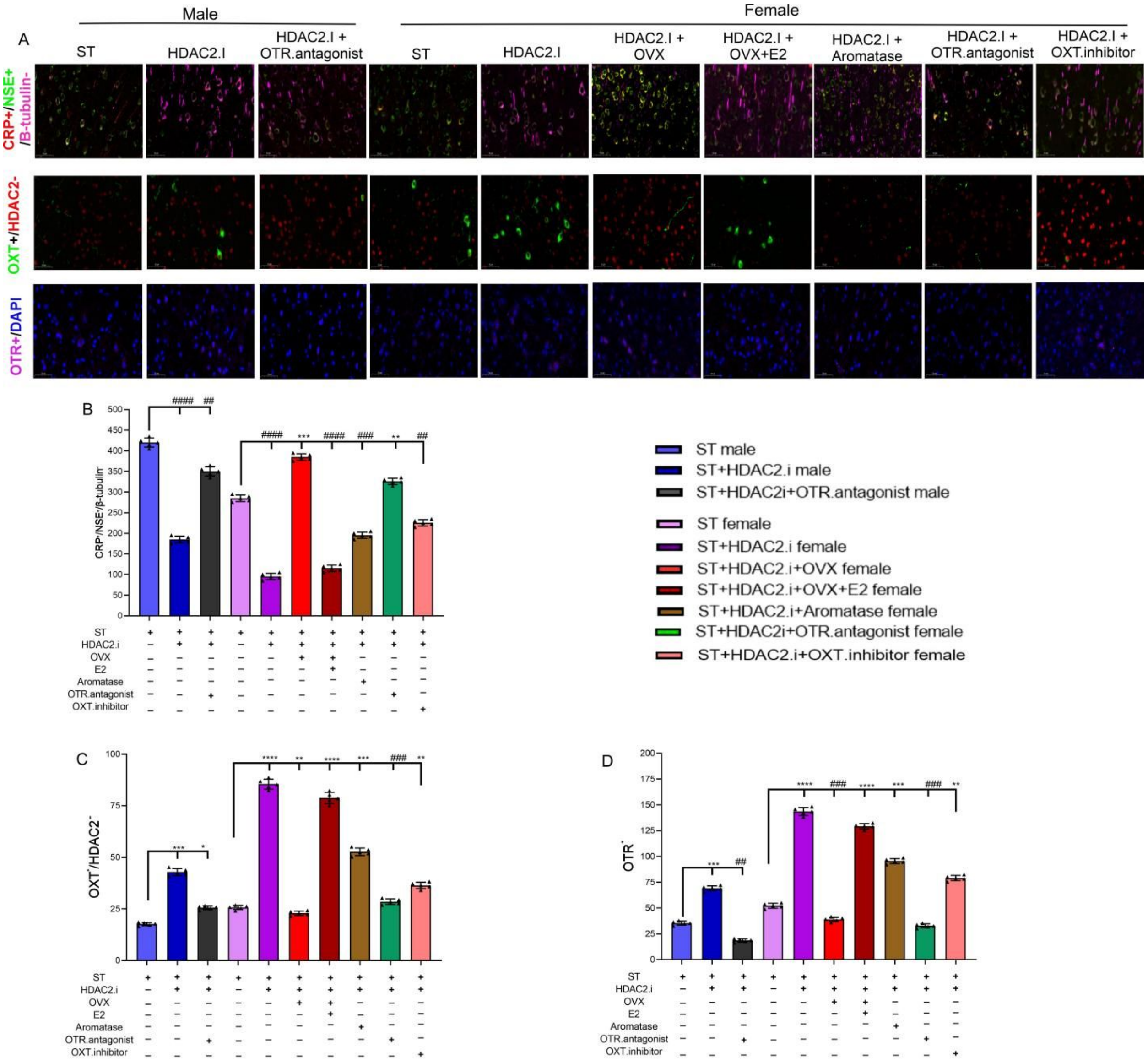

Figure S5. Estrogen and oxytocin signaling modulate cytokine levels and the OXT/HDAC2 axis following HDAC2 inhibition. A. Representative immunofluorescence images of triple staining CRP/NSE/B-tubulin, double OXT/HDAC2, single OTR staining. **B.** $CRP^{+}NSE^{+}B\text{-tubulin}^{+}$ cells. **C.** OXT^{+} $HDAC2^{+}$ cells number across all experimental groups, representing the balance between neuroprotective oxytocin signaling and epigenetic repression.**D**. OTR^{+} cells, n=5 Data are presented as mean ± SEM. Ovariectomy (OVX) or aromatase inhibition abolishes the protective effects of HDAC2.i in females, increasing CRP^{+} NSE^{+} $B\text{-tubulin}^{+}$ cells and decreasing the OXT^{+} $HDAC2^{+}$ cells number(♀ST+HDAC2.i vs. ♀ST+HDAC2.i+OVX). Estradiol replacement (E2) completely rescues the protective phenotype in OVX females. Blockade of the oxytocin receptor (OTR.antagonist) or oxytocin release (OXT.inhibitor) reverses HDAC2.i-mediated protection in females, increasing cytokines and decreasing the OXT/HDAC2 ratio (♀ST+HDAC2.i vs. ♀ST+HDAC2.i+OTR.antagonist).

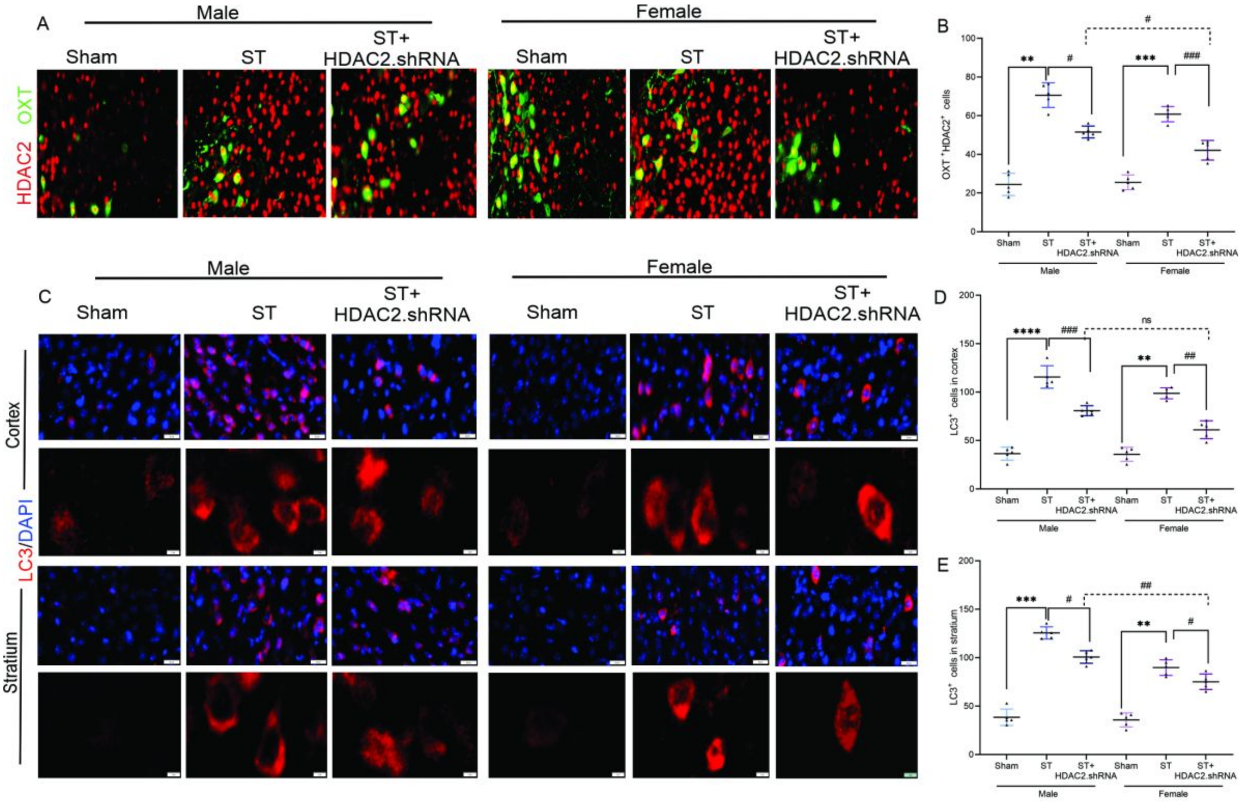

Figure S6. Validation of viral transduction efficiency and analysis of autophagic markers following HDAC2 knockdown. Data confirm effective knockdown of HDAC2 in both sexes following AAV9-shRNA injection. **A.** Representative immunofluorescence images of HDAC2 /OXT double immunostaining. **B**. OXT$^+$ HDAC2$^+$ cells, ♀ST+HDAC2.shRNA vs. ♂ST+HDAC2.shRNA; $^{\#}p<0.05$, **C.** Representative immunofluorescence images of LC3 staining (autophagic marker) in the cortex and stratium of male and female mice from Sham, ST, and ST+HDAC2.shRNA groups. Scale bar = 50 μm.. **D**. LC3$^+$ cells in the cortex, ♀ST+HDAC2.shRNA vs. ♂ST+HDAC2.shRNA,; p=ns, **E**. LC3$^+$ cells in the striatum, ♀ST+HDAC2.shRNA vs. ♂ST+HDAC2.shRNA;$^{\#\#\#}p<0.01$. n=5, Data expressed as SEM±., n=5, Data expressed as SEM±. These data confirm effective and equivalent HDAC2 knockdown in both sexes and demonstrate that HDAC2 regulates post-stroke autophagy in a sexually dimorphic manner, with females showing greater attenuation of pathological autophagic activity.

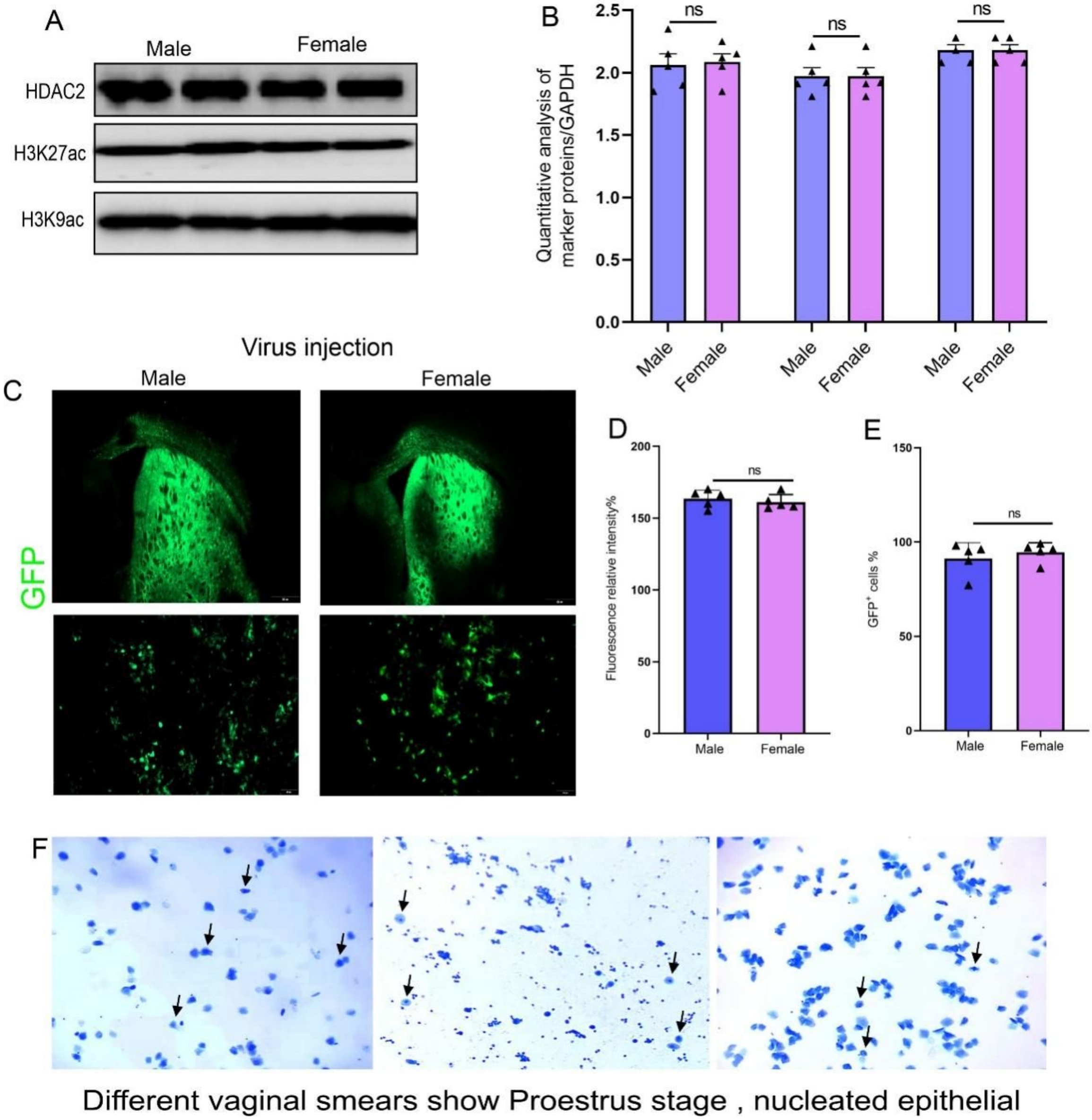

Figure.S7 Validation of viral transduction efficiency, drug delivery, and estrous cycle monitoring. **A.** Western blot analysis comparing protein expression of the histone deacetylase HDAC2 and the histone acetylation marks H3K27ac and H3K9ac in brain homogenates from male and female mice 2 hr after apicidin injection. **B.** Quantification of proteins normalized to GAPDH ; *p=ns*. , n=5 Data expressed as mean ± SEM. **C.** Representative immunofluorescence images showing GFP expression in the target region (CPu) of male and female mice 3 weeks post-injection, confirming successful viral transduction. Scale bar = 100 μm.. **D.** Analysis of mean GFP fluorescence intensity per cell; *p=ns*. **E.** Analysis of the proportion of transduced (GFP+) cells within the region of interest ♂ST+HDAC2.shRNA vs. ♀ST+HDAC2.shRNA; *p=ns*. **F.**Representative images of vaginal cytology smears from female mice, showing the distinct cell types used to determine estrous cycle stage. For all primary

endpoint experiments involving stroke surgery, behavioral testing, and tissue collection, female mice were exclusively used in the proestrus stage, characterized by a predominance of nucleated epithelial cells. This standardization controls for hormonally-driven variability in oxytocin receptor expression and stroke vulnerability.

Supplementary Tables

Table. S1. Antibodies used in this study

Antibody	Supplier	Cat. No.	Host
HDAC2	Novus Bio	5113T	Mouse
HSP90	Cell signaling technology	4877	Rabbit
SMN	Abclonal	A16246	Rabbit
OTR	Cell signaling technology	3033	Rabbit
OXT	Abcam	Ab212193	Rabbit
CREB	Cell signaling technology	9197	Rabbit
BDNF	Abcam	EPR1292	Rabbit
TrkB	Protein-tech	29961-1-AP	Rabbit
SYN I	Abcam	ab254349	Rabbit
iNOS	Cell signaling technology	13120	Rabbit
nNOS	Cell signaling technology	4231	Rabbit
eNOS	Cell signaling technology	32027	Rabbit
Bax	Abclonal	A19684	Rabbit
Bcl-2	Abclonal	A20777	Rabbit
c-caspase3	Cell signaling technology	9661	Rabbit
IL6	Abclonal	A0286	Rabbit
TNF-α	Abclonal	A11534	Rabbit
GAPDH	Cell Signaling Technology	177487	Mouse
NeuN	Cell Signaling Technology	94403	Mouse
MAP2	Cell signaling technology	32536	Mouse

P53	Protein-tech	60283-2-Ig	Rabbit
Cyto-C	Cell signaling technology	12963	Mouse
Tomm20	Cell signaling technology	Ab186735	Rabbit
CRP	Protein-tech	66250-1-Ig	Rabbit
NSE	Protein-tech	10149-1-AP	Rabbit
ATG5	Abclonal	A0203	Rabbit
Beclin1	Abclonal	A21191	Rabbit
LC3	Abclonal	A5618	Rabbit
GFAP	Cell Signaling Technology	3670	Mouse
Iba-1	Cell signaling technology	17198	Rabbit
AQP4	Abcam	ab259318	Rabbit
acetyl h3-lys27	Cell signaling technology	8173T	Rabbit
acetyl h3-lys9	Cell signaling technology	9649T	Rabbit

Table.S2 List of genes within 4 groups (Con-M, ST-M, Con-F,ST-F)

Cont-M	St-M	Cont-F	St-F
Ddx3y	Ddx3y	Aqp4	Xist
Xist	Kdm5d	Bpifa6	Ddx3y
Kdm5d	Lgals3bp	Ndn	Kdm5d
Uty	Xist	Psapl1	Ndn
Gm14308	Uty	BC048679	Uty
Eif2s3y	Gm14308	Hopx	Nnt
Gm29650	Eif2s3y	Aadacl4fm1	Eif2s3y
Psapl1	Cldn11	Ppia	Gfap
Srp54a	C3	Elovl3	Cnp
BC048679	Marcksl1	Ugt1a8	Eif2s3x
Lgals3bp	Gm29650	Aadac	Cldn11
Aadacl4fm1	Ctsd	Gm21982	Trf
Ugt1a8	9630013A20Rik	Pck1	Lgals3bp
Pcdha11	Cnp	Aqp8	Mag
C4b	Six3os1	Gm17494	Apoe
Cst7	Fam107a	Gm20431	Aqp4

Aadac	Gfap	S100a5	Gm29650
Fam177a	H2-D1	E230020A03Rik	Ddx3x
Gm20431	Thbs4	Esp6	Pitpnm2
Pck1	C1qb	Cyp4a10	Gm42047
Aqp8	Tspan2	Cyp2b10	Ctsd
Lag3	Fosb	Gata6os	Cd9
Nnt	H2-K1	Gm17167	C1qb
Esp6	Pcsk1	Gm5859	Pllp
Eif2s3x	Eif2s3x	Aadacl3	Lag3
Gfap	Tnk2	Aadacl4fm4	Gm20458
Lbhd2	B2m	Ctse	Gm20390
Cyp4a10	Cd9	Gm9944	Map1a
Aqp3	B3gnt2	Irx2	A2m
Cyp2b10	Lag3	Tmigd1	Arpc1b
Gata6os	C1qa	Gm9451	Rps18-ps4
Aadacl3	Mog	Cldn7	Gm49378
Ctse	Gm49388	Kcnk15	B2m
Aadacl4fm4	Gm42047	Gm44501	Etnppl
Gm9944	C1qc	Adig	Gm45837
Tmigd1	Apoe	Srp54a	Gbp4
Gm9451	Gm20458	Il20	Pknox2
Cldn7	Lcn2	Cyp4a32	Rnasek
Gm44501	Syt4	Aadacl4	Sparc
Adig	Clec7a	Mgam	Mbp
Il20	Habp4	Gm16136	Slc36a2
Cyp4a32	Iqsec3	1810019D21Rik	Dbp
Aadacl4	Mt2	Cyp2j8	Vim
Gm49320	Git1	Ces1e	Tspan2
Gm16136	Gm10076	Foxd3	Zfp973
Hopx	Gm10736	Upk3b	Serpina3n
Rps18-ps4	Etnppl	Elk1	Ifi27l2a
Ces1e	C1ql2	Zfp91	Mog
Tmem139	Arpc1b	Pllp	Fgr
Gm49380	Pcdha11	Gdpd4	Camk2a
Gm20390	Dennd5b	Lbhd2	Map2
Foxd3	Adcy1	Igf2	H2-D1
Gm20458	Nptx2	Ednrb	Ago2
Upk3b	Gsn	Kcnn4	Grin2a
B2m	Pcdhgb1	Slc16a7	Siglecf
Zfp973	Ddx3x	Slc38a3	Ifit3
Gm45837	Oasl2	Rbp1	Cnksr3

Lox	Panx2	Gm10599	Prr32
Fam177a2	Tmem151b	Siglecf	Igf2
Gm44502	Egr3	Pisd-ps1	Efcab9
Srp54b	1700047M11Rik	Gm17081	Fam107a
H2-Aa	Gm37827	Clec4d	Lilrb4a
B4gat1	Gm49486	6430553K19Rik	Ncor2
A2m	A2m	Gm26673	Kif5a
Ctsd	Peg10	Zic1	Enc1
A230004M16Rik	Gpr34	Igfbpl1	Gm17167
Vnn1	Rbm3	Slc36a2	Lcn2
Papln	Igsf9b	Glp1r	Shox2
Gdpd3	Tmcc2	Sntb1	Gm5859
Peg10	Ctss	Sox1ot	Shank2
Clec4d	Pitpnm2	Lrp4	Cobl
Zfp91	Aifm3	Fam131c	Homer2
Ramp2	Mag	Tmem215	Pdzd2
Fras1	Rims3	Atf4	Plp1
Naip1	Map2	Rab38	Pcdhgc3
Ddx3x	Irf7	Fam177a	Mt2
A2ml1	Aldh1a1	Itpka	Spred1
Dsp	Il1rl1	Hmgcs2	Elk1
Tube1	Gbp3	Gm7808	9330159F19Rik
Sox1ot	Lox	Gm26782	Gins2
Uba1y	Ttbk1	Mgst1	Sipa1l1
H2-D1	Cenpf	Gm44544	9630013A20Rik
Prg4	Mpeg1	Zkscan3	Gm26782
Etnppl	Scd1	Mmp14	Mpdz
Spata18	Gm44502	Rab8b	Nrep
Ifit3	Gfod1	Gm47403	Homer1
Vim	Stac2	Alox12b	Gm21093
Six1	Pcdhga2	AI606473	Peg10
Ankrd24	Homer2	Slc35d3	Trim9
H2-Q4	Mal	Smtnl2	Gata3
Cplx3	Scrt1	Pax6	Arhgdib
Gm50323	Cxcl10	Rapgef4	H2-Q4
Clec7a	Ramp2	Gm42047	Cd63
AI606473	Sirt2	Oxt	Mrtfb
Homer2	Trem2	Irs4	Npc2
Oxt	Oxt	Sgk1	Rasgrf2
Pax6	Pax6	Th	Enpp6
Irs4	Irs4	Gfap	Hectd4

Sgk1	Sgk1	Vim	Gm3883
Th	Th	Kdm5d	Oxt
Lrp4	Vim	Xist	Pax6
Gh	Lrp4	Gh	Irs4
Igf2	Gh	Mbp	Sgk1
Mbp	Igf2	Cnp	Th
Cnp	Mbp	Plp1	Lrp4
Plp1	Plp1	B2m	Gh

Table.S3 Genes with HDAC2 connections

Gene	Relation to HDAC2	Evidence/Function
Gfap (astrocyte marker)	Indirect	HDAC2 regulates astrocyte activation
Mbp (myelin basic protein)	Indirect	HDAC2 modulates myelination
Plp1	Indirect	HDAC inhibitors regulates oligodendrocyte function
Cnp (myelin-associated gene)	Indirect	modulates myelination
Pax6	Indirect	HDAC2 regulates Pax6 in neurodevelopment
Igf2	Indirect	HDAC2 modulates Igf2 imprinting and expression.
Btg2	Indirect	HDAC2 represses *Btg2* (cell cycle regulator).
Th (Tyrosine Hydroxylase)	Indirect	HDAC2 affects dopaminergic neuron gene regulation.
Vim (Vimentin)	Indirect	HDAC2 regulates cytoskeletal genes in cancer/glia.
Fabp7	Indirect	HDAC2 modulates fatty acid metabolism in neurons.

Table.S4 Genes with OXT connections

Gene	Relation to OXT	Evidence/Function

Oxt(Oxytocin)	Direct	The gene itself encodes oxytocin
Gh(Growth Hormone)	Indirect	OXT stimulates Gh release in the pituitary
Sgk1 (Serum/Glucocorticoid-Regulated Kinase 1)	Indirect	OXT signaling modulates stress responses via Sgk1
Irs4 (Insulin Receptor Substrate 4)	Indirect	OXT influences metabolic signaling in the hypothalamus
Vipr2(Vasoactive Intestinal Peptide Receptor 2)	Indirect	Interacts with neuropeptide systems linked to OXT
Gal (Galanin)	Indirect	Co-expressed with OXT in hypothalamic neurons.
Cartpt(CART Prepropeptide)	Indirect	Involved in appetite regulation, modulated by OXT.
Tsc22d3(Glucocorticoid-Induced Leucine Zipper)	Indirect	OXT interacts with stress-related glucocorticoid pathways

Table.S5 List of proteins predicted as possible direct targets

**Target name**	**Confidence**	**ChEMBL id**
Histone deacetylase 1	0.9321	CHEMBL325
Histone deacetylase 3	0.6881	CHEMBL1829
Histone deacetylase 2	0.6291	CHEMBL1937
Endothelin receptor ET-A	0.5391	CHEMBL252
P-glycoprotein 1	0.442	CHEMBL4302
Histone deacetylase 8	0.3982	CHEMBL3192
MAP kinase ERK1	0.2715	CHEMBL3385
Oxytocin receptor	0.1768	CHEMBL2049
Somatostatin receptor 5	0.1516	CHEMBL1792
Histone deacetylase 6	0.1338	CHEMBL1865
Melanocortin receptor 5	0.1112	CHEMBL4608

Somatostatin receptor 2	0.0988	CHEMBL1804
Histone deacetylase 11	0.0938	CHEMBL3310
Histone deacetylase 4	0.0907	CHEMBL3524
Motilin receptor	0.0897	CHEMBL2203
Neurokinin 1 receptor	0.0825	CHEMBL249
Somatostatin receptor 1	0.0791	CHEMBL1917
Histone deacetylase 10	0.0782	CHEMBL5103
Somatostatin receptor 4	0.0742	CHEMBL1853
Urotensin II receptor	0.0508	CHEMBL3764
Vasopressin V1a receptor	0.0397	CHEMBL1889
Baculoviral IAP repeat-containing protein 3	0.023	CHEMBL5335
TNF-alpha	0.0222	CHEMBL1825
Appetite-regulating hormone	0.0176	CHEMBL1921664
Growth factor receptor-bound protein 2	0.0163	CHEMBL3663
Melanocortin receptor 3	0.0154	CHEMBL4644
Vasopressin V2 receptor	0.0113	CHEMBL1790
Menin	0.0108	CHEMBL1615381
Neurokinin 2 receptor	0.0022	CHEMBL2327

Table.S6 List of proteins predicted as possible targets

**Target name**	**Confidence**	**ChEMBL id**
Histone deacetylase	0.835	CHEMBL2093865
Tyrosine-protein kinase FYN	0.5123	CHEMBL1841
Histone deacetylase 1	0.3161	CHEMBL325
Histone deacetylase 3/Nuclear receptor corepressor 2 (HDAC3/NCoR2)	0.3028	CHEMBL2111363
Histone deacetylase 8	0.2527	CHEMBL3192
Vasopressin V1a receptor	0.2416	CHEMBL1889

Thromboxane-A synthase	0.213	CHEMBL1835
Histone deacetylase 3	0.1458	CHEMBL1829
Melanocortin receptor 5	0.1274	CHEMBL4608
P-glycoprotein 1	0.1071	CHEMBL4302
Oxytocin receptor	0.095	CHEMBL2049
Somatostatin receptor 1	0.0669	CHEMBL1917
Somatostatin receptor 2	0.0604	CHEMBL1804
Somatostatin receptor 4	0.0475	CHEMBL1853
Histone deacetylase 4	0.0466	CHEMBL3524
Melanocortin receptor 1	0.046	CHEMBL3795
Somatostatin receptor 5	0.0422	CHEMBL1792
Melanocortin receptor 3	0.0415	CHEMBL4644
Histone deacetylase 3/NCoR1	0.0382	CHEMBL3038484
Histone deacetylase 6	0.0358	CHEMBL1865
Somatostatin receptor 3	0.028	CHEMBL2028
Appetite-regulating hormone	0.022	CHEMBL1921664
Solute carrier organic anion transporter family member 1B1	0.0199	CHEMBL1697668
Vasopressin V2 receptor	0.0158	CHEMBL1790
Histone deacetylase 2	0.012	CHEMBL1937
Splicing factor 3B subunit 3	0.0111	CHEMBL1250378
Integrin alpha-V/beta-5	0.0012	CHEMBL2096675
